# Social play experience in juvenile rats is indispensable for appropriate socio-sexual behavior in adulthood in males but not females

**DOI:** 10.3389/fnbeh.2022.1076765

**Published:** 2023-01-23

**Authors:** Ashley E. Marquardt, Jonathan W. VanRyzin, Rebeca W. Fuquen, Margaret M. McCarthy

**Affiliations:** ^1^Program in Neuroscience, University of Maryland School of Medicine, Baltimore, MD, United States; ^2^Department of Pharmacology, University of Maryland School of Medicine, Baltimore, MD, United States

**Keywords:** behavior, social play, play, social isolation, social behavior, sex differences

## Abstract

Social play is a dynamic and rewarding behavior abundantly expressed by most mammals during the juvenile period. While its exact function is debated, various rodent studies on the effects of juvenile social isolation suggest that participating in play is essential to appropriate behavior and reproductive success in adulthood. However, the vast majority of these studies were conducted in one sex only, a critical concern given the fact that there are known sex differences in play’s expression: across nearly all species that play, males play more frequently and intensely than females, and there are qualitative sex differences in play patterns. Further limiting our understanding of the importance of play is the use of total isolation to prevent interactions with other juveniles. Here, we employed a novel cage design to specifically prevent play in rats while allowing for other forms of social interaction. We find that play deprivation during the juvenile period results in enduring sex-specific effects on later-life behavior, primarily in males. Males prevented from playing as juveniles exhibited decreased sexual behavior, hypersociability, and increased aggressiveness in adulthood, with no effects on these measures in females. Importantly, play deprivation had no effect on anxiety-like behavior, object memory, sex preference, or social recognition in either sex, showing the specificity of the identified impairments, though there were overall sex differences in many of these measures. Additionally, acute play deprivation impaired performance on a test of prosocial behavior in both sexes, indicating a difference in the motivation and/or ability to acquire this empathy-driven task. Together, these findings provide novel insight into the importance and function of juvenile social play and how this differs in males and females.

## Introduction

Broadly seen in most mammalian species from rodents to humans, social play (also known as rough-and-tumble play or play-fighting) has fascinated neuroscientists and evolutionary biologists alike for decades. Though animals spend upwards of 20% of their time participating in play during adolescence (Pellis and Pellis, 2009), this well-conserved behavior serves no obvious purpose, yet appears fundamental for appropriate development. Various studies have found that juvenile social isolation in rats leads to impairments in social behavior (Hol et al., 1999; Van den Berg et al., 1999; Von Frijtag et al., 2002), cognition (Einon et al., 1978; Baarendse et al., 2013; Yusufishaq and Rosenkranz, 2013), and sexual behavior (Gerall et al., 1967; Cooke et al., 2000), increases anxiety- and depression-like behavior (Parker and Morinan, 1986; Wright et al., 1991; Arakawa, 2003; Leussis and Andersen, 2008; Lukkes et al., 2009; Cuesta et al., 2020), and impacts susceptibility to addiction-related behaviors (Whitaker et al., 2013; Baarendse et al., 2014).

While these studies provide insight on the impacts of juvenile social isolation writ large, there are three important caveats that preclude the ability to apply them to assess the importance of social play specifically. First, isolation prevents *all* social interaction—both play and non-play—so it is unclear in most of these studies whether and to what extent any identified impairments are due to the lack of play vs. the lack of general social interaction, as well as the additional stress that total isolation induces (Begni et al., 2020). Second, in many of these studies, animals were isolated through adulthood, when testing was conducted; as such, it is also unclear whether and to what extent there may be an effect of acute isolation on the observed phenotypes. Finally, and most notably, the majority of these studies were conducted in only one sex—typically, male subjects—thereby preventing the ability to assess whether there are sex differences in the identified impairments.

This final caveat is of critical importance given there are robust sex differences in social play that are seemingly as well-conserved as play itself. Across nearly all species that play, from rodents to humans, male juveniles play more frequently and intensely than females (see VanRyzin et al., 2020a for review). Additionally, there are known sex differences in the qualitative characteristics of social play interactions (Pellis and Pellis, 1990, 1997). Studies investigating the neural underpinnings of this behavioral sex difference have identified various nodes within the larger circuitry of social behavior, including the medial amygdala (VanRyzin et al., 2019) and lateral septum (Bredewold et al., 2014), which exert sex-specific influences on play following sexual differentiation early in life as part of typical brain development. Indeed, deficits in play are core symptoms of neurodevelopmental disorders like autism spectrum disorder, attention-deficit/hyperactivity disorder, and early onset schizophrenia (Alessandri, 1992; Jones et al., 1994; Møller and Husby, 2000; Jordan, 2003; Helgeland and Torgersen, 2005), many of which also exhibit robust sex differences in diagnosis and symptomology (Aleman et al., 2003; Ramtekkar et al., 2010; Arnett et al., 2015; Halladay et al., 2015; Giordano et al., 2021; Prosperi et al., 2021). The robust and conserved nature of this sex difference, then, speaks to its importance and begs the question: does play serve a different purpose in males compared to females?

**Figure 1:**
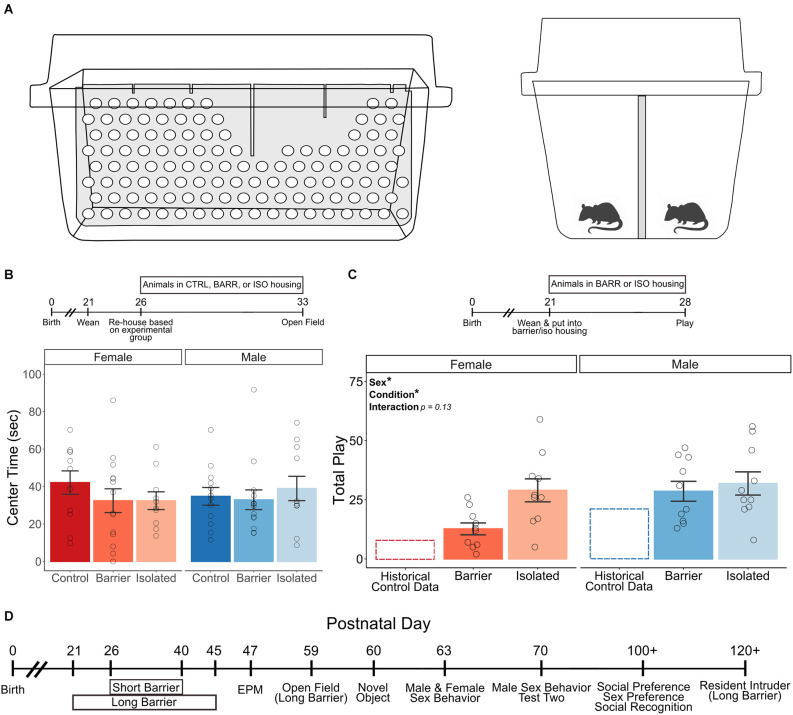
Experimental approach and assessment of acute effects of barrier separation/isolation. Cartoons depicting the perforated Plexiglass cage divider (in light gray) used to prevent play in BARR animals **(A)**, viewed from the side (left panel, no animals shown) and straight on (right panel). Acute play deprivation has no effect on anxiety-like behavior as evidenced by center time in an open field test **(B)**, with timeline of experiment shown above graph); additionally, the ability and motivation to engage in play is maintained (**C**, showing the intact play in BARR and ISO animals of both sexes compared to historical control data, with timeline of experiment shown above graph). A timeline of experimental procedures for the remainder of the study is shown in **(D)**. All behavioral tests shown on the timeline were conducted in both the short and long duration barrier studies unless otherwise noted. Bars indicate group means ± SEM, and open circles represent data from individual rats. *p < 0.05, *n* = 10–14 per group.

Here, we investigate the impact of juvenile play experience on later-life behaviors and how this may differ as a consequence of sex. Juvenile rats of both sexes were deprived of play *via* one of two different methods or were housed under normal conditions, i.e., controls. For the first play deprivation method, we created perforated Plexiglass cage dividers (“play barriers”) that could be placed into standard home cages to physically separate the two juvenile rats inside (Figure 1A). Improving upon the previous methodology, this manipulation prevents play while still allowing for other forms of social interaction in the visual, auditory, olfactory, and tactile domain. Interaction with a conspecific across a similar physical barrier has been shown to be socially rewarding (Peartree et al., 2012) and to reduce anxiety-like behavior as compared to full isolation, an additional benefit (Klapper-Goldstein et al., 2020). For the second play deprivation method, we eliminated all social interactions (play and non-play) by socially isolating animals *via* single-housing, as done by others. Animals were placed in these or control housing conditions as juveniles, then re-housed in standard group housing around puberty, after which we assessed the impact on various adult behaviors. Supporting our hypothesis, we found that social play experience impacts later-life endpoints in a sex-specific manner. Play deprivation preferentially impacted behaviors within the socio-sexual domain, decreasing sexual behavior, increasing sociability, and increasing aggressiveness in adulthood in males but not females, providing valuable insight into sex differences in the function of this fundamental adolescent behavior.

## Materials and Methods

### Animals and housing conditions

Adult Sprague-Dawley rats (Charles River Laboratories, Wilmington, MA, USA) were maintained on a 12:12 h reverse light-dark cycle with food and water available *ad libitum*. Animals were mated in our facility and allowed to deliver normally under standard laboratory conditions, with the day of birth designated as postnatal day 0 (P0). Both male and female pups were used, with condition groups and sexes balanced across multiple litters. Rats were weaned on P21 in same-sex, sibling pairs and housed in polycarbonate cages (20 × 40 × 20 cm) with corncob bedding. For the short duration barrier (SDB) experiments, rats either remained in control housing conditions or were subjected to one of two play deprivation groups from P26–40: barrier separation (BARR) or full social isolation (ISO). On P26, a thin, transparent Plexiglass cage divider (Total Plastics, Baltimore, MD, USA; approximately 45 × 21 × 0.5 cm) containing 98 evenly spaced 1.5 cm diameter holes were inserted into the middle of the home cage of BARR rats. This Plexiglass barrier created two identical compartments within the home cage and served to separate the pair of animals and therefore prevent them from physically engaging in social play behavior, while still allowing for visual, olfactory, auditory, and tactile communication between the two. In contrast, on P26, ISO rats were placed alone in a new standard cage and subjected to full social isolation. Control rats continued to be housed in pairs for the full extent of this time period. Animals remained in these conditions until P40, when BARR and ISO animals were re-housed in the same same-sex, sibling pairs as before, under standard housing conditions. For the long duration barrier (LDB) experiments, the same experimental procedures were applied, except animals were placed in the appropriate housing conditions for a longer period: from P21 (upon weaning) through P45. A total of 196 rats were used across all experiments. All experimental procedures were approved by the Institutional Animal Care and Use Committee’s regulations at the University of Maryland School of Medicine.

### Behavioral testing

All behavioral testing was performed during the dark phase of the light-dark cycle under red light illumination. Unless stated otherwise, all behavioral tests were performed and scored offline by an experimenter blind to condition (at all times) and sex (when appropriate).

#### Social play (P28)

In a separate cohort of animals used for initial experiments, subjects were individually placed with a sex- and age-matched, control-housed stimulus animal into an enclosure (49 × 37 cm, 24 cm high) with TEK-Fresh cellulose bedding (Envigo, Indianapolis, IN, USA). Only BARR and ISO animals were used in this experiment, as the goal was to assess whether the motivation and/or ability to play was maintained in animals despite the altered housing conditions. Animals were allowed to acclimate to the arena for 2 min, then video recorded for 10 min. Videos were manually scored offline to quantify the number of pounces, pins, and boxing behaviors, summed together as the “total play” exhibited in the test. Further detail on the scoring parameters for each of the three assessed play behaviors can be found in VanRyzin et al. (2020b).

#### Open field (P33 or P59)

Rats were individually placed in an open polycarbonate arena (78 × 78 cm, 40 cm high) underlaid with a grid delineating perimeter and center regions and video-recorded for 10 min. Videos were manually scored offline for the number of gridline crossings and time spent in the center region of the arena.

#### Elevated plus maze (P47)

Rats were individually placed in the center of a black polycarbonate plus maze consisting of two open (102.5 × 12 cm) and two closed (102.5 × 12 cm, 45.5 cm high) opposing arms, elevated 72 cm from the ground. Rats were allowed to explore the maze for 5 min while automatically recorded using a video camera and ANY-maze video tracking software (Stoelting, Wood Dale, IL, USA) to determine the percentage of the test duration spent in the open arms of the maze and the total distance traveled.

#### Novel object recognition (P60)

Rats were individually placed in the same open polycarbonate arena used for the open field test for 5 min and allowed to investigate a pair of identical objects placed on opposite ends of the arena. Following this initial exposure, rats were placed back in their home cages. One hour later, rats were returned to the arena, where they were exposed to the now familiar object and a novel object, again on opposite ends of the arena. Videos were recorded during both sessions and manually scored offline using a virtual stopwatch for the time spent investigating each object on the second test, from which the discrimination ratio [(time with novel object − time with familiar object)/(time with novel object + time with familiar object)] was calculated. The position of objects within the arena and the order of object exposure was counterbalanced across groups.

#### Female sex behavior (P63)

For the SDB study, intact female rats were hormonally primed ahead of the sexual behavior test by receiving subcutaneous injections of 10 μg of estradiol benzoate (Millipore Sigma, St. Louis, MO, USA) in 0.1 ml sesame oil (Millipore Sigma) 1 and 2 days before testing (P61 and P62, respectively), and 500 μg of progesterone (Millipore Sigma) in 0.1 ml sesame oil 6 h before testing on P63. For the LDB study, female subjects were allowed to naturally cycle without any hormonal priming. Vaginal smears were taken daily to determine the estrus cycle stage, and females were tested on the day of proestrus.

For the sex behavior test itself, females were placed in an enclosure (49 × 37 cm, 24 cm high) with TEK-Fresh cellulose bedding (Envigo, Indianapolis, IN, USA) with an adult male stimulus rat for 10 min. Videos were manually scored for the number of lordoses in response to a mount by the stimulus male and the number of proceptive behaviors (number of hops, darts, and solicitations) as previously described (VanRyzin et al., 2016).

#### Male sex behavior (P63 and P70)

Intact male rats were tested for expression of copulatory behaviors in response to a hormonally primed female stimulus rat. Two tests of male sex behavior took place: one on P63, and another 1 week later on P70. For both tests, stimulus females were hormonally primed as described above, with subcutaneous injections of estradiol benzoate 1 and 2 days before and progesterone 6 h before testing. For the sex behavior tests, males were placed in an enclosure as described above with a primed adult female stimulus rat for 20 min. Videos were manually scored offline for the number of mounts, intromissions, and ejaculations as previously described (VanRyzin et al., 2016). The refractory period, or the resting period following an ejaculation before the male rat began exhibiting mounts and intromissions again, was also recorded and used to determine the active time (full time of the 20 min test − length of the refractory period). From this, each animal’s mount rate (the total number of mounts, intromissions, and ejaculations divided by the active time and multiplied by 60 to get the mounts per minute) was calculated for each test, as well as the percentage of animals in each group that ejaculated on one or both tests.

#### Social preference (P100+)

For the social preference test, a two-chambered open-topped polycarbonate apparatus (100 × 50 cm, 35 cm high) was used. In the corner of one chamber (“social chamber”), a novel same-sex adult (P60+) stimulus rat was placed under a small (20 × 20 × 20 cm) clear polycarbonate box (“stimulus box”) containing small 1.25 cm holes to allow the test rat to see, hear, smell, and have some tactile interactions with the stimulus rat. In the corner of the other chamber (“empty chamber”), an identical clear polycarbonate box was placed without a stimulus rat. Test rats were individually placed in the neutral zone of this apparatus and allowed to freely explore for 5 min while video recorded and automatically tracked using ANY-maze software. The time spent in each chamber and the time spent nearby (within 5 cm, deemed the “interaction zone”) each stimulus box was recorded, and the percentage of time near the social box (time spent in the interaction zone of the social chamber/time spent in the interaction zone of the social chamber + time spent in the interaction zone of the empty chamber) was calculated, as well as the ratio of time spent near the social chamber to time spent near the empty chamber. The position of the social chamber and the empty chamber within the arena was counterbalanced across groups.

#### Sex preference (P100+)

Using the same apparatus and procedure as described above for the social preference test, the sex preference test assessed the amount of time a test rat spent interacting with a same-sex vs. an opposite-sex stimulus animal. In this test, one chamber of the apparatus contained a novel adult (P60+) male rat under the stimulus box, while the other chamber contained a novel adult female rat under the stimulus box. As before, test rats were individually placed in the neutral zone of the apparatus and allowed to freely explore for 5 min while video recorded and automatically tracked using ANY-maze. From this, the percentage of time spent closely interacting with the opposite-sex stimulus animal (time spent in the interaction zone of the opposite-sex chamber/time spent in the interaction zone of the opposite-sex chamber + time spent in the interaction zone of the same-sex chamber) was calculated, as well as the ratio of time spent near the opposite-sex chamber to time spent near the same-sex chamber. The position of the chamber containing the male stimulus animal and the chamber containing the female stimulus animal within the arena was counterbalanced across groups.

#### Social recognition (P100+)

To allow for habituation and increase social motivation, rats were singly housed in a test cage identical to their home cage for 2 h before the start of the test. After 2 h, a novel same-sex juvenile (P24–30) stimulus rat was placed into the test cage with the test rat for 5 min (“Train” trial) and their interactions were video-recorded. After 5 min, the stimulus rat was removed, and the test rat remained in the test cage for a retention interval of 30 min, after which the same stimulus rat (now familiar) was placed back in the test cage. Interactions between the test rat and the stimulus rat were again recorded for 5 min (“Test” trial). Videos were manually scored offline for the time spent by the test rat investigating the stimulus rat in both tests, and the ratio of the time spent interacting with the stimulus rat on the Test trial (familiar) compared to the Train trial (novel) was calculated. Separately, a control experiment was independently conducted which followed the same procedure; however, a novel stimulus rat was placed in the cage with the test rat in both the Train trial and the Test trial, to control for any effects of a second interaction trial in general, unrelated to recognition of the stimulus animal or the lack thereof.

#### Resident intruder assay (P120+)

We assessed aggressive behavior in the resident-intruder assay in adult males using a procedure modified from Koolhaas et al. (2013). Males were isolated in their home cages for 48 h prior to the start of the test. On the test day, a novel, smaller (weighing >150 g less than the test animal) stimulus male was placed into the cage with the test male. Their interactions were recorded for 15 min, after which the stimulus male was removed from the test animal’s home cage. After a 30 min inter-trial interval, the test was repeated with a novel stimulus male. Videos were manually scored offline for the following behaviors: keep downs, in which the test animal pins down the stimulus animal by placing its front paws on the chest of the stimulus; lateral threats, in which the test animal pushes its flank towards the stimulus; upright postures, in which both animals stand up on their hind legs and grasp at each other’s front legs; mounting behavior; and overall clinch attacks. The total number of aggressive behaviors displayed in each test was calculated.

#### Empathy/prosocial helping behavior

We assessed empathy behavior using a paradigm modified from Kight et al. (2021). Rats of both sexes were weaned on P21 into CTRL or BARR housing conditions, as described above. On P26, animals were individually placed in an enclosure (49 × 37 cm, 24 cm high) with TEK-Fresh cellulose bedding (Envigo, Indianapolis, IN, USA) containing an empty and open 14 × 8 × 9 cm clear polycarbonate confinement box and allowed to explore for 10 min to habituate to the box and arena. This confinement box contained a hinged door that is blocked by a lever when closed, requiring the animal outside the confinement box to push the lever open in order for the animal inside the confinement box to freely escape. On P27, one animal from a sex- and condition-matched cagemate pair was placed inside the confinement box. A funnel filled with ice was positioned over the confinement box, dripping cool water inside and thus motivating the animal to seek release. Video recording and a count-up timer began when the other member of the cagemate pair (the test subject) was placed in the arena. If at any time the test subject released their cagemate from the confinement box, the recording was stopped and the time of release was recorded. Animals were allowed to interact for 10 s, then swiftly returned to CTRL or BARR housing conditions. If 15 min elapsed without release, the lever blocking the door was loosened slightly by the experimenter (turned 90 degrees such that there was no gap in the door but that the lever no longer blocked the exit) to facilitate learning. If an additional 5 min passed (20 min total testing time), the recording was stopped and the door opened further to allow for free exit. In this case, the time of release was denoted as 20 min (the maximum). This procedure was repeated once daily until P38, with each member of the cagemate pair serving the same role each day (i.e., there was no switching as to which member of the pair was the test subject vs. the confined subject).

### Statistical analysis

Specific values for test statistics, *p* values, and effect sizes are listed in Table 1 and referenced in the text, when appropriate. Statistical analysis was performed using RStudio (RStudio Team, 2021; version 1.4.1106) and GraphPad Prism (GraphPad Software, San Diego, CA, USA; version 7.04). All data were initially analyzed with a Shapiro-Wilk normality test to determine if data were normally distributed. Data which were normally distributed were then analyzed with a two-way ANOVA with factors for sex and juvenile housing condition (CTRL, BARR, or ISO) unless otherwise stated. If a significant main effect of housing condition or a significant interaction was detected, *post hoc* analysis was conducted using a Tukey’s honestly significant difference test to determine which groups differed. If a significant main effect of condition was detected in cases in which a one-way ANOVA was conducted (i.e., tests in which sex was not a factor, such as male sex behavior), *post hoc* analysis was conducted using Fisher’s least significant difference procedure, as there were three groups (Meier, 2006). In some cases (Novel Object Recognition, Social Preference, and Sex Preference tests), one-sample *t*-tests were used to determine whether group means differed from chance; additionally, paired *t*-tests were used to compare the Train trial to the Test trial for the Social Recognition test. Data which were not normally distributed were analyzed using Kruskal-Wallis tests or Wilcoxon rank sum tests when appropriate, as described in Table 1. Analyses were considered significant when *p* < 0.05.

**Table 1:** Summary of statistical parameters.

Test	Data structure	Type of test	Description of analysis	Test value	*p*-value	Effect size
Open Field—Acute Deprivation						
Center Time	Non-normal	Wilcoxon rank-sum test	Effect of sex	*W* = 657	0.919	*r* = 0.0126
	Non-normal	Kruskal-Wallis test: males	Effect of condition	*χ*^2^_(2)_ = 0.893	0.64	*η*^2^_(H)_ = −0.033
	Non-normal	Kruskal-Wallis test: females	Effect of condition	*χ*^2^_(2)_ = 1.9	0.387	*η*^2^_(H)_ = −0.003
Play—Acute Deprivation						
Total Play	**Normal distribution**	**Two-way ANOVA**	**Main effect: sex**	***F*_(1,36)_ = 4.929**	**0.036**	*η*^2^_(H)_ = −0.101
	**Normal distribution**	**Two-way ANOVA**	**Main effect: condition**	***F*_(1,36)_ = 5.358**	**0.026**	*η*^2^_(H)_ = −0.11
	Normal distribution	Two-way ANOVA	Interaction: sex × condition	*F*_(1,36)_ = 2.357	0.134	*η*^2^_(H)_ = 0.048
Elevated Plus Maze—SDB						
Time in Open Arms (%)	Normal distribution	Two-way ANOVA	Main effect: sex	*F*_(1,70)_ = 2.835	0.097	*η*^2^ = 0.038
	Normal distribution	Two-way ANOVA	Main effect: condition	*F*_(2,70)_ = 1.330	0.271	*η*^2^ = 0.037
	Normal distribution	Two-way ANOVA	Interaction: sex × condition	*F*_(2,70)_ = 0.107	0.899	*η*^2^ = 0.003
Distance Traveled	**Normal distribution**	**Two-way ANOVA**	**Main effect: sex**	***F*_(1,70)_ = 4.704**	**0.034**	***η*^2^ = 0.065**
	Normal distribution	Two-way ANOVA	Main effect: condition	*F*_(2,70)_ = 0.183	0.833	*η*^2^ = 0.005
	Normal distribution	Two-way ANOVA	Interaction: sex × condition	*F*_(2,70)_ = 0.912	0.406	*η*^2^ = 0.025
Elevated Plus Maze—LDB						
Time in Open Arms (%)	**Normal distribution**	**Two-way ANOVA**	**Main effect: sex**	***F*_(1,62)_ = 7.654**	**0.007**	***η*^2^ = 0.106**
	Normal distribution	Two-way ANOVA	Main effect: condition	*F*_(2,62)_ = 3.038	0.055	*η*^2^ = 0.089
	Normal distribution	Two-way ANOVA	Interaction: sex × condition	*F*_(2,62)_ = 0.149	0.862	*η*^2^ = 0.005
Distance Traveled	**Normal distribution**	**Two-way ANOVA**	**Main effect: sex**	***F*_(1,62)_ = 8.595**	**0.005**	***η*^2^ = 0.113**
	**Normal distribution**	**Two-way ANOVA**	**Main effect: condition**	***F*_(2,62)_ = 10.326**	**<0.001**	***η*^2^ = 0.250**
		***Two-way ANOVA: Tukey post-hoc***	***CTRL vs. BARR***	***n/a***	**<0.001**	***d* = 1.655**
		*Two-way ANOVA: Tukey post-hoc*	*CTRL vs. ISO*	*n/a*	0.544	*d* = 0.298
		***Two-way ANOVA: Tukey post-hoc***	***BARR vs. ISO***	***n/a***	**0.003**	***d* = 0.937**
	Normal distribution	Two-way ANOVA	Interaction: sex × condition	*F*_(2,62)_ = 0.239	0.788	*η*^2^ = 0.008
Open Field Test—LDB						
Center Time	**Non-normal**	**Wilcoxon rank-sum test**	**Effect of sex**	***W* = 1,080.5**	**<0.001**	***r* = 0.428**
	Non-normal	Kruskal-Wallis test: males	Effect of condition	*χ*^2^_(2)_ = 0.024	0.988	*d* = −0.055
	Non-normal	Kruskal-Wallis test: females	Effect of condition	*χ*^2^_(2)_ = 0.234	0.89	*d* = −0.052
Line Crossings	**Normal distribution**	**Two-way ANOVA**	**Main effect: sex**	***F*_(1,70)_ = 19.673**	**<0.001**	**η2 = 0.221**
	**Normal distribution**	**Two-way ANOVA**	**Main effect: condition**	***F*_(2,70)_ = 4.649**	**0.013**	***η*^2^ = 0.117**
		***Two-way ANOVA: Tukey post-hoc***	***CTRL vs. BARR***	***n/a***	**0.01**	***d* = 0.709**
		*Two-way ANOVA: Tukey post-hoc*	*CTRL vs. ISO*	*n/a*	0.117	*d* = 0.509
		*Two-way ANOVA: Tukey post-hoc*	*BARR vs. ISO*	*n/a*	0.512	*d* = 0.311
	Normal distribution	Two-way ANOVA	Interaction: sex × condition	*F*_(2,70)_ = 0.777	0.464	*η*^2^ = 0.022
Novel Object—SDB						
**Discrimination Ratio**	**Normal distribution**	**One sample *t*-test**	**Male CTRL vs. 0**	***t*_(11)_ = 3.369**	**0.006**	***d* = 0.972**
	**Normal distribution**	**One sample *t*-test**	**Male BARR vs. 0**	***t*_(13)_ = 4.537**	**<0.001**	***d* = 1.213**
	**Normal distribution**	**One sample *t*-test**	**Male ISO vs. 0**	***t*_(12)_ = 3.437**	**0.005**	***d* = 0.953**
	**Normal distribution**	**One sample *t*-test**	**Female CTRL vs. 0**	***t*_(13)_ = 2.822**	**0.014**	***d* = 0.754**
	**Normal distribution**	**One sample *t*-test**	**Female BARR vs. 0**	***t*_(13)_ = 3.322**	**0.006**	***d* = 0.888**
	**Normal distribution**	**One sample *t*-test**	**Female ISO vs. 0**	***t*_(10)_ = 3.932**	**0.003**	***d* = 1.186**
	Normal distribution	Two-way ANOVA	Main effect: sex	*F*_(1,72)_ = 0.421	0.519	*η*^2^ = 0.008
	Normal distribution	Two-way ANOVA	Main effect: condition	*F*_(2,72)_ = 1.334	0.27	*η*^2^ = 0.036
	Normal distribution	Two-way ANOVA	Interaction: sex × condition	*F*_(2,72)_ = 0.634	0.533	*η*^2^ = 0.017
Novel Object—LDB						
Discrimination Ratio	**Normal distribution**	**One sample *t*-test**	**Male CTRL vs. 0**	***t*_(10)_ = 2.631**	**0.025**	***d* = 0.793**
	**Normal distribution**	**One sample *t*-test**	**Male BARR vs. 0**	***t*_(8)_ = 3.806**	**0.005**	***d* = 1.269**
	**Normal distribution**	**One sample *t*-test**	**Male ISO vs. 0**	***t*_(14)_ = 3.943**	**0.001**	***d* = 1.018**
	**Normal distribution**	**One sample *t*-test**	**Female CTRL vs. 0**	***t*_(10)_ = 4.947**	**<0.001**	***d* = 1.492**
	**Normal distribution**	**One sample *t*-test**	**Female BARR vs. 0**	***t*_(11)_ = 3.541**	**0.005**	***d* = 1.022**
	**Normal distribution**	**One sample *t*-test**	**Female ISO vs. 0**	***t*_(12)_ = 0.001**	**0.003**	***d* = 1.165**
	Normal distribution	Two-way ANOVA	Main effect: sex	*F*_(1,65)_ = 0.756	0.388	*η*^2^ = 0.013
	Normal distribution	Two-way ANOVA	Main effect: condition	*F*_(2,65)_ = 0.416	0.661	*η*^2^ = 0.013
	Normal distribution	Two-way ANOVA	Interaction: sex × condition	*F*_(2,65)_ = 0.723	0.489	*η*^2^ = 0.022
Female Sex Behavior—SDB						
Lordosis Quotient	Non-normal	Kruskal-Wallis test	Effect of condition	*χ*^2^_(2)_ = 3.257	0.196	*η*^2^_(H)_ = 0.034
Proceptive Behaviors	Normal distribution	One-way ANOVA	Effect of condition	*F*_(2,37)_ = 0.137	0.873	*η*^2^ = 0.007
Female Sex Behavior—LDB						
Lordosis Quotient	Non-normal	Kruskal-Wallis test	Effect of condition	*χ*^2^_(2)_ = 3.52	0.172	*η*^2^_(H)_ = 0.056
Proceptive Behaviors	Non-normal	Kruskal-Wallis test	Effect of condition	*χ*^2^_(2)_ = 1.61	0.447	*η*^2^_(H)_ = −0.014
Male Sex Behavior—SDB						
Average Number of Mounts	Normal distribution	One-way ANOVA	Effect of condition	*F*_(2,35)_ = 2.786	0.075	0.216
Average Number of Intromissions	Normal distribution	One-way ANOVA	Effect of condition	*F*_(2,35)_ = 2.165	0.13	0.137
Average Mount Rate	**Normal distribution**	**One-way ANOVA**	**Effect of condition**	***F*_(2,35)_ = 4.818**	**0.014**	**0.11**
		***Kruskal-Wallis: Fisher’s LSD post-hoc***	***CTRL vs. BARR***	***n/a***	**0.03**	***d* = 0.618**
		***Kruskal-Wallis: Fisher’s LSD post-hoc***	***CTRL vs. ISO***	***n/a***	**0.005**	***d* = 0.981**
		*Kruskal-Wallis: Fisher’s LSD post-hoc*	*BARR vs. ISO*	n/a	0.407	*d* = 0.221
Percent that Ejaculated	Non-normal	Chi-square goodness of fit test	% ejaculated vs. 90% - CTRL	*χ*^2^ = 0.818	0.366	φ= 0.273
		Chi-square goodness of fit test	% ejaculated vs. 90% - BARR	*χ*^2^ = 0.127	0.722	φ= 0.095
		Chi-square goodness of fit test	% ejaculated vs. 90% - ISO	*χ*^2^ = 0.419	0.518	φ= 0.18
Male Sex Behavior—LDB						
Average Number of Mounts	Normal distribution	**One-way ANOVA**	**Effect of condition**	***F*_(2,33)_ = 3.949**	**0.029**	***η*^2^ = 0.193**
		***One-way ANOVA: Fisher’s LSD post-hoc***	***CTRL vs. BARR***	***n/a***	**0.03**	***d* = 0.654**
		***One-way ANOVA: Fisher’s LSD post-hoc***	***CTRL vs. ISO***	***n/a***	**0.014**	***d* = 0.626**
		*One-way ANOVA: Fisher’s LSD post-hoc*	*BARR vs. ISO*	*n/a*	0.767	*d* = 0.06
Average Number of Intromissions	Non-normal	**Kruskal-Wallis test**	**Effect of condition**	***χ*^2^_(2)_ = 8.304**	**0.016**	*η*^2^_(H)_ = 0.191
		***Kruskal-Wallis: Fisher’s LSD post-hoc***	***CTRL vs. BARR***	***n/a***	**0.026**	***d* = 0.796**
		***Kruskal-Wallis: Fisher’s LSD post-hoc***	***CTRL vs. ISO***	***n/a***	**0.012**	***d* = 0.906**
		*Kruskal-Wallis: Fisher’s LSD post-hoc*	*BARR vs. ISO*	n/a	0.763	*d* = 0.182
Average Mount Rate	Non-normal	Kruskal-Wallis test	Effect of condition	*χ*^2^_(2)_ = 3.779	0.151	*η*^2^_(H)_ = 0.054
Percent that Ejaculated	Non-normal	Chi-square goodness of fit test	% ejaculated vs. 90% - CTRL	*χ*^2^ = 0.056	0.814	**φ** = 0.084
		**Chi-square goodness of fit test**	**% ejaculated vs. 90% - BARR**	***χ*^2^ = 10**	**0.002**	***φ* = 1**
		**Chi-square goodness of fit test**	**% ejaculated vs. 90% - ISO**	***χ*^2^ = 11.701**	**<0.001**	***φ* = 0.949**
Social Preference—SDB						
Percent of Time Near Social Box	**Normal distribution**	**One sample *t*-test**	**Male CTRL vs. 50%**	***t*_(11)_ = 8.363**	**<0.001**	***d* = 2.414**
	**Normal distribution**	**One sample *t*-test**	**Male BARR vs. 50%**	***t*_(13)_ = 19.047**	**<0.001**	***d* = 5.091**
	**Normal distribution**	**One sample *t*-test**	**Male ISO vs. 50%**	***t*_(12)_ = 6.702**	**<0.001**	***d* = 1.859**
	**Normal distribution**	**One sample *t*-test**	**Female CTRL vs. 50%**	***t*_(13)_ = 10.423**	**<0.001**	***d* = 2.786**
	**Normal distribution**	**One sample *t*-test**	**Female BARR vs. 50%**	***t*_(13)_ = 7.674**	**<0.001**	***d* = 2.051**
	**Normal distribution**	**One sample *t*-test**	**Female ISO vs. 50%**	***t*_(11)_ = 6.631**	**<0.001**	***d* = 1.914**
	**Normal distribution**	**Two-way ANOVA**	**Main effect: sex**	***F*_(1,73)_ = 18.582**	**<0.001**	***η*^2^ = 0.142**
	**Normal distribution**	**Two-way ANOVA**	**Main effect: condition**	***F*_(2,73)_ = 8.168**	**<0.001**	***η*^2^ = 0.126**
	**Normal distribution**	**Two-way ANOVA**	**Interaction: sex × condition**	***F*_(2,73)_ = 10.672**	**<0.001**	***η*^2^ = 0.165**
		*Two-way ANOVA: Tukey post-hoc*	*Male CTRL vs. Female CTRL*	n/a	0.778	*d* = 0.579
		*Two-way ANOVA: Tukey post-hoc*	*Female BARR vs. Female CTRL*	n/a	1	*d* = 0.006
		***Two-way ANOVA: Tukey post-hoc***	***Male BARR vs. Female CTRL***	**n/a**	**<0.001**	***d* = 2.939**
		*Two-way ANOVA: Tukey post-hoc*	*Female ISO vs. Female CTRL*	n/a	0.99	*d* = 0.248
		*Two-way ANOVA: Tukey post-hoc*	*Male ISO vs. Female CTRL*	n/a	0.999	*d* = 0.147
		*Two-way ANOVA: Tukey post-hoc*	*Female BARR vs. Male CTRL*	n/a	0.786	*d* = 0.501
		***Two-way ANOVA: Tukey post-hoc***	***Male BARR vs. Male CTRL***	**n/a**	**<0.001**	***d* = 1.911**
		*Two-way ANOVA: Tukey post-hoc*	*Female ISO vs. Male CTRL*	n/a	0.984	*d* = 0.247
		*Two-way ANOVA: Tukey post-hoc*	*Male ISO vs. Male CTRL*	n/a	0.936	*d* = 0.247
		***Two-way ANOVA: Tukey post-hoc***	***Male BARR vs. Female BARR***	**n/a**	**<0.001**	***d* = 2.515**
		*Two-way ANOVA: Tukey post-hoc*	*Female ISO vs. Female BARR*	n/a	0.991	*d* = 0.215
		*Two-way ANOVA: Tukey post-hoc*	*Male ISO vs. Female BARR*	n/a	0.999	*d* = 0.126
		***Two-way ANOVA: Tukey post-hoc***	***Female ISO vs. Male BARR***	**n/a**	**<0.001**	***d* = 2.056**
		***Two-way ANOVA: Tukey post-hoc***	***Male ISO vs. Male BARR***	**n/a**	**<0.001**	***d* = 2.156**
		*Two-way ANOVA: Tukey post-hoc*	*Male ISO vs. Female ISO*	n/a	1	*d* = 0.082
Ratio Time Near Social Box	**Non-normal**	**Wilcoxon rank-sum test**	**Effect of sex**	***W* = 449**	**0.001**	***r* = 0.365**
	**Non-normal**	**Kruskal-Wallis test: males**	**Effect of condition**	***χ*^2^_(2)_ = 19.374**	**<0.001**	*η*^2^_(H)_ = 0.483
		***Kruskal-Wallis: Wilcoxon post-hoc***	***Male CTRL vs. BARR***	***W* = 15**	**<0.001**	***r* = 0.696**
		*Kruskal-Wallis: Wilcoxon post-hoc*	*Male CTRL vs. ISO*	*W* = 98	0.295	*r* = 0.218
		***Kruskal-Wallis: Wilcoxon post-hoc***	***Male BARR vs. ISO***	***W* = 170**	**<0.001**	***r* = 0.738**
	Non-normal	Kruskal-Wallis test: females	Effect of condition	*χ*^2^_(2)_ = 0.557	0.757	*η*^2^_(H)_ = −0.039
Social Preference—LDB						
Percent of Time Near Social Box	**Normal distribution**	**One sample *t*-test**	**Male CTRL vs. 50%**	***t*_(11)_ = 6.639**	**<0.001**	***d* = 1.917**
	**Normal distribution**	**One sample *t*-test**	**Male BARR vs. 50%**	***t*_(11)_ = 6.794**	**<0.001**	***d* = 1.961**
	**Normal distribution**	**One sample *t*-test**	**Male ISO vs. 50%**	***t*_(10)_ = 3.837**	**0.003**	***d* = 1.157**
	**Normal distribution**	**One sample *t*-test**	**Female CTRL vs. 50%**	***t*_(11)_ = 5.178**	**<0.001**	***d* = 1.495**
	**Normal distribution**	**One sample *t*-test**	**Female BARR vs. 50%**	***t*_(11)_ = 5.547**	**<0.001**	***d* = 1.601**
	**Normal distribution**	**One sample *t*-test**	**Female ISO vs. 50%**	***t*_(12)_ = 2.520**	**0.027**	***d* = 0.699**
	**Normal distribution**	**Two-way ANOVA**	**Main effect: sex**	***F*_(1,66)_ = 9.471**	**0.003**	***η*^2^ = 0.121**
	Normal distribution	Two-way ANOVA	Main effect: condition	*F*_(2,66)_ = 1.192	0.31	*η*^2^ = 0.03
	Normal distribution	Two-way ANOVA	Interaction: sex × condition	*F*_(2,66)_ = 0.239	0.788	*η*^2^ = 0.006
Ratio Time Near Social Box	**Non-normal**	**Wilcoxon rank-sum test**	**Effect of sex**	***W* = 354**	**<0.001**	***r* = 0.390**
	Non-normal	Kruskal-Wallis test: males	Effect of condition	*χ*^2^_(2)_ = 0.134	0.935	*η*^2^_(H)_ = −0.058
	Non-normal	Kruskal-Wallis test: females	Effect of condition	*χ*^2^_(2)_ = 2.51	0.285	*η*^2^_(H)_ = 0.015
Sex Preference—SDB						
Percent Time Near Opposite-Sex Box	**Normal distribution**	**One sample *t*-test**	**Male CTRL vs. 50%**	***t*_(11)_ = 3.953**	**0.002**	***d* = 1.141**
	**Normal distribution**	**One sample *t*-test**	**Male BARR vs. 50%**	***t*_(13)_ = 4.009**	**0.001**	***d* = 1.071**
	**Normal distribution**	**One sample *t*-test**	**Male ISO vs. 50%**	***t*_(12)_ = 2.423**	**0.032**	***d* = 0.672**
	**Normal distribution**	**One sample *t*-test**	**Female CTRL vs. 50%**	***t*_(13)_ = 2.355**	**0.035**	***d* = 0.629**
	**Normal distribution**	**One sample *t*-test**	**Female BARR vs. 50%**	***t*_(13)_ = 3.007**	**0.01**	***d* = 0.804**
	**Normal distribution**	**One sample *t*-test**	**Female ISO vs. 50%**	***t*_(11)_ = 3.558**	**0.004**	***d* = 1.027**
	Normal distribution	Two-way ANOVA	Main effect: sex	*F*_(1,73)_ = 0.037	0.849	*η*^2^ = 0.001
	Normal distribution	Two-way ANOVA	Main effect: condition	*F*_(2,73)_ = 0.559	0.574	*η*^2^ = 0.015
	Normal distribution	Two-way ANOVA	Interaction: sex × condition	*F*_(2,73)_ = 0.996	0.374	*η*^2^ = 0.026
Ratio Time Near Opposite-Sex Box	Non-normal	Wilcoxon rank-sum test	Effect of sex	*W* =795	0.887	*r* = 0.017
	Non-normal	Kruskal-Wallis test: males	Effect of condition	*χ*^2^_(2)_ = 3.334	0.189	*η*^2^_(H)_ = 0.037
	Non-normal	Kruskal-Wallis test: females	Effect of condition	*χ*^2^_(2)_ = 0.017	0.992	*η*^2^_(H)_ = −0.054
Sex Preference—LDB						
Percent Time Near Opposite-Sex Box	**Normal distribution**	**One sample *t*-test**	**Male CTRL vs. 50%**	***t*_(11)_ = 3.313**	**0.007**	***d* = 0.956**
	Normal distribution	One sample *t*-test	Male BARR vs. 50%	*t*_(11)_ = 1.966	0.075	*d* = 0.567
	**Normal distribution**	**One sample *t*-test**	**Male ISO vs. 50%**	***t*_(11)_ = 2.339**	**0.04**	***d* = 0.675**
	**Normal distribution**	**One sample *t*-test**	**Female CTRL vs. 50%**	***t*_(11)_ = 4.881**	**<0.001**	***d* = 1.409**
	Normal distribution	One sample *t*-test	Female BARR vs. 50%	*t*_(11)_ = 1.557	0.148	*d* = 0.449
	**Normal distribution**	**One sample *t*-test**	**Female ISO vs. 50%**	***t*_(12)_ = 4.034**	**0.002**	***d* = 1.119**
	Normal distribution	Two-way ANOVA	Main effect: sex	*F*_(1,67)_ = 0.741	0.392	*η*^2^ = 0.011
	Normal distribution	Two-way ANOVA	Main effect: condition	*F*_(2,67)_ = 0.811	0.449	*η*^2^ = 0.023
	Normal distribution	Two-way ANOVA	Interaction: sex × condition	*F*_(2,67)_ = 1.167	0.317	*η*^2^ = 0.033
Ratio Time Near Opposite-Sex	Non-normal	Wilcoxon rank-sum test	Effect of sex	*W* = 595	0.437	*r* = 0.092
	Non-normal	Kruskal-Wallis test: males	Effect of condition	*χ*^2^_(2)_ = 1.5	0.472	*η*^2^_(H)_ = −0.015
	Non-normal	Kruskal-Wallis test: females	Effect of condition	*χ*^2^_(2)_ = 5.059	0.08	*η*^2^_(H)_ = 0.09
Interaction Time	**Normal distribution**	**Paired *t*-test**	**Male CTRL-Train vs. Test**	***t*_(12)_ = 2.336**	**0.038**	***d* = 0.592**
	Normal distribution	Paired *t*-test	Male BARR-Train vs. Test	*t*_(11)_ = 1.892	0.085	*d* = 0.518
	**Normal distribution**	**Paired *t*-test**	**Male ISO-Train vs. Test**	***t*_(12)_ = 2.491**	**0.028**	***d* = 0.746**
	**Normal distribution**	**Paired *t*-test**	**Female CTRL-Train vs. Test**	***t*_(9)_ = 4.316**	**0.002**	***d* = 1.276**
	**Normal distribution**	**Paired *t*-test**	**Female BARR-Train vs. Test**	***t*_(8)_ = 2.692**	**0.027**	***d* = 1.055**
	**Normal distribution**	**Paired *t*-test**	**Female ISO-Train vs. Test**	***t*_(8)_ = 4.791**	**0.001**	***d* = 1.455**
Ratio of Investigation Duration	**Non-normal**	**Wilcoxon rank-sum test**	**Effect of sex**	***W* = 253**	**<0.001**	*r* = 0.446
	Non-normal	Kruskal-Wallis test: males	Effect of condition	*χ*^2^_(2)_ = 0.03	0.985	*η*^2^_(H)_ = −0.056
	Non-normal	Kruskal-Wallis test: females	Effect of condition	*χ*^2^_(2)_ = 0.22	0.896	*η*^2^_(H)_ = −0.072
Social Recognition-LDB						
Interaction Time	**Normal distribution**	**Paired *t*-test**	**Male CTRL-Train vs. Test**	***t*_(11)_ = 2.653**	**0.022**	***d* = 0.713**
	**Normal distribution**	**Paired *t*-test**	**Male BARR-Train vs. Test**	***t*_(11)_ = 2.425**	**0.033**	***d* = 0.707**
	**Normal distribution**	**Paired *t*-test**	**Male ISO-Train vs. Test**	***t*_(11)_ = 3.019**	**0.012**	***d* = 0.629**
	**Normal distribution**	**Paired *t*-test**	**Female CTRL-Train vs. Test**	***t*_(11)_ = 6.737**	**<0.001**	***d* = 1.861**
	**Normal distribution**	**Paired *t*-test**	**Female BARR-Train vs. Test**	***t*_(11)_ = 10.557**	**<0.001**	***d* = 3.492**
	**Normal distribution**	**Paired *t*-test**	**Female ISO-Train vs. Test**	***t*_(12)_ = 5.839**	**<0.001**	***d* = 1.477**
Ratio of Investigation Duration	**Non-normal**	**Wilcoxon rank-sum test**	**Effect of sex**	***W* = 110**	**<0.001**	***r* = 0.718**
	Non-normal	Kruskal-Wallis test: males	Effect of condition	*χ*^2^_(2)_ = 0.126	0.939	*η*^2^_(H)_ = −0.057
	Non-normal	Kruskal-Wallis test: females	Effect of condition	*χ*^2^_(2)_ = 3.552	0.169	*η*^2^_(H)_ = 0.046
Resident Intruder Assay-LDB						
Average Number of Keep Downs	Normal distribution	One-way ANOVA	Effect of condition	*F*_(2,27)_ = 1.773	0.189	*η*^2^ = 0.116
Average Number of Lateral Threats	Non-normal	Kruskal-Wallis test	Effect of condition	*χ*^2^_(2)_ = 4.08	0.13	*η*^2^_(H)_ = 0.077
Average Number of Upright Postures	Non-normal	Kruskal-Wallis test	Effect of condition	*χ*^2^_(2)_ = 4.856	0.088	*η*^2^_(H)_ = 0.106
Average Number of Clinch Attacks	Non-normal	Kruskal-Wallis test	Effect of condition	*χ*^2^_(2)_ = 0.799	0.671	*η*^2^_(H)_ = −0.045
Average Number of Aggressive Behaviors (Total)	**Normal distribution**	**One-way ANOVA**	**Effect of condition**	***F*_(2,27)_ = 4.815**	**0.016**	***η*^2^ = 0.263**
		*One-way ANOVA: Fisher’s LSD post-hoc*	*CTRL vs. BARR*	*n/a*	0.056	*d* = 0.863
		***One-way ANOVA: Fisher’s LSD post-hoc***	***CTRL vs. ISO***	***n/a***	**0.005**	***d* = 1.481**
		*One-way ANOVA: Fisher’s LSD post-hoc*	*BARR vs. ISO*	*n/a*	0.30	*d* = 0.456
Empathy/Prosocial Helping Behavior						
Average Mean Latency to Release Cagemate—Days 1–6	**Normal distribution**	**Two-way ANOVA**	**Main effect: sex**	***F*_(1,15)_ = 4.757**	**0.046**	***η*^2^ = 0.217**
	**Normal distribution**	**Two-way ANOVA**	**Main effect: condition**	***F*_(1,15)_ = 6.049**	**0.027**	***η*^2^ = 0.287**
	Normal distribution	Two-way ANOVA	Interaction: sex × condition	*F*_(1,15)_ = 0.157	0.697	*η*^2^ = 0.01
Average Mean Latency to Release Cagemate—Days 7–12	Normal distribution	Two-way ANOVA	Main effect: sex	*F*_(1,15)_ = 4.028	0.063	*η*^2^ = 0.217
	Normal distribution	Two-way ANOVA	Main effect: condition	*F*_(1,15)_ = 0.344	0.566	*η*^2^ = 0.022
	Normal distribution	Two-way ANOVA	Interaction: sex × condition	*F*_(1,15)_ = 0.99	0.336	*η*^2^ = 0.062
Factor Analysis–SDB						
Factor Scores—Anxiety	Normal distribution	One-way ANOVA	Effect of condition	*F*_(2,20)_ = 0.439	0.651	*η*^2^ = 0.042
Factor Scores—Salience	**Normal distribution**	**One-way ANOVA**	**Effect of condition**	***F*_(2,20)_ = 4.029**	**0.034**	***η*^2^ = 0.287**
		***One-way ANOVA: Fisher’s LSD post-hoc***	***CTRL vs. BARR***	***n/a***	**0.0285**	***d* = 1.521**
		***One-way ANOVA: Fisher’s LSD post-hoc***	***CTRL vs. ISO***	***n/a***	**0.015**	***d* = 1.33**
		*One-way ANOVA: Fisher’s LSD post-hoc*	*BARR vs. ISO*	*n/a*	0.797	*d* = 0.119
Factor Scores—Sociosexual Behavior	**Normal distribution**	**One-way ANOVA**	**Effect of condition**	***F*_(2,20)_ = 3.674**	**0.044**	***η*^2^ = 0.269**
		***One-way ANOVA: Fisher’s LSD post-hoc***	***CTRL vs. BARR***	***n/a***	**0.019**	***d* = 1.265**
		*One-way ANOVA: Fisher’s LSD post-hoc*	*CTRL vs. ISO*	*n/a*	0.447	*d* = 0.55
		*One-way ANOVA: Fisher’s LSD post-hoc*	*BARR vs. ISO*	*n/a*	0.059	*d* = 0.877
Factor Scores—Anxiety I	Normal distribution	One-way ANOVA	Effect of condition	*F*_(2,20)_ = 0.537	0.593	*η*^2^ = 0.051
Factor Scores—Anxiety II	Normal distribution	One-way ANOVA	Effect of condition	*F*_(2,20)_ = 3.306	0.058	*η*^2^ = 0.248
Factor Scores—Sociosexual Behavior	**Normal distribution**	**One-way ANOVA**	**Effect of condition**	***F*_(2,20)_ = 4.196**	**0.03**	***η*^2^ = 0.296**
		***One-way ANOVA: Fisher’s LSD post-hoc***	***CTRL vs. BARR***	***n/a***	**0.009**	***d* = 1.706**
		*One-way ANOVA: Fisher’s LSD post-hoc*	*CTRL vs. ISO*	*n/a*	0.167	*d* = 0.746
		*One-way ANOVA: Fisher’s LSD post-hoc*	*BARR vs. ISO*	*n/a*	0.147	*d* = 0.683

#### Factor analysis

We chose *a priori* to conduct factor analysis on the Short Duration Barrier and Long Duration Barrier studies separately, to determine whether the identified factors would be similar or different across the two experiments. Only subjects that had complete datasets were included in the factor analysis. Data were normalized by log transformation, then initially analyzed for adequacy for factor analysis using the Kaiser-Meyer-Olkin factor adequacy test and Bartlett’s test of sphericity for the correlation matrix of the dataset. In both the SDB and LDB studies, female data was deemed insufficiently correlated and therefore inadequate for factor analysis based on these indices (Kaiser-Meyer-Olkin MSA below 0.5 for both studies; Bartlett’s sphericity *p* > 0.05). For this reason, we conducted a factor analysis on only the male data, a choice supported by the fact that the vast majority of our observed effects of play deprivation were seen in males but not females. Using male data alone, datasets from both the SDB and LDB study indicated appropriateness for factor analysis, with an overall Kaiser-Meyer-Olkin MSA above 0.5 (SDB: MSA = 0.53; LDB: MSA = 0.53) and a significant *p-value* for Bartlett’s test (SDB: *p* = 0.001; LDB: *p* < 0.001). This resulted in an overall *n* of 23 for both studies (SDB: *n* = 6 for CTRL; *n* = 8 for BARR; *n* = 9 for ISO. LDB: *n* = 8 for CTRL; *n* = 7 for BARR; *n* = 8 for ISO).

Factor analysis was then conducted using varimax rotation with a factor-loading cutoff of 0.5. The resulting factors were retained if their eigenvalue was greater than 1 (based on the Kaiser criterion), which generated a three-factor solution for both the SDB and LDB datasets. Behavioral variables which had low communality (below 0.3) and did not load onto any of the three factors above the factor loading cutoff of 0.3 were removed, and analysis was repeated using the parameters described above, again identifying a three-factor solution for both datasets. We then calculated individual factor scores for each subject and analyzed these scores for an effect of condition independently by factor using a one-way ANOVA, as described further above.

## Results

To ensure our manipulation did not induce overt behavioral pathology on its own, we began by assessing the acute effects of play deprivation. During the juvenile age (at either P26 or P21 for the open field and playfulness tests, respectively), same-sex sibling pairs of rats of both sexes were placed into control (CTRL) or barrier (BARR) cages or were single-housed in isolation cages (ISO). One week later, we assessed anxiety-like behavior *via* the open field test. In a separate cohort of BARR and ISO animals, we also assessed playfulness. We found no effect of sex or housing condition on center time in the open field (Figure 1B). As seen in previous studies of juvenile isolation (Panksepp and Beatty, 1980; Ikemoto and Panksepp, 1992), we found that the ability and motivation to participate in play was not only intact in BARR and ISO animals but was increased relative to our historical data for control-housed animals (Figure 1C; Argue and McCarthy, 2015). Interestingly, when we assessed the BARR and ISO play data, we detected a significant main effect of both sex (*p* = 0.036) and treatment (*p* = 0.026), with males of both housing conditions and ISO animals of both sexes playing significantly more than females and BARR animals, respectively. However, this effect of the condition seems to be driven more strongly in females than in males. Although the sex × condition interaction did not reach statistical significance (*p* = 0.13), the mean difference in play levels between female BARR and ISO animals (12.7 vs. 29.0 play events) is much larger than that of male BARR and ISO animals (28.6 vs. 31.9 play events). This suggests that the increased social interaction allowed by the BARR housing condition is sufficient to reduce play motivation in females but not males, an unexpected finding speaking to the differential role that play may serve in females compared to males. Overall, these initial studies indicate that the modified housing used to prevent play does not in itself increase anxiety-like behavior or impair the ability to play if given the opportunity and provides intriguing evidence in support of the idea that play may serve different purposes across the sexes.

For adult behavioral experiments, rats were weaned on P21 into same-sex sibling pairs. In the short duration barrier experiments (SDB), rats were then placed into barrier or isolation cages starting on P26 or remained in control housing conditions (Figure 1D). Animals remained in these conditions until P40, a 2-week period encompassing the peak of the window in which social play is maximally expressed (Panksepp, 1981), after which they were returned to standard group housing for the remainder of the experiments. To determine whether there were any sex differences in resiliency to play deprivation, we repeated our behavioral assays in the long duration barrier experiments (LDB). In this study, animals were placed in control, barrier, or isolation conditions for as long as could be tolerated based on size: starting at weaning (P21) and lasting until P45, an age when rats of both sexes have typically entered puberty, at which point playfulness dramatically decreases (Meaney and Stewart, 1981; Panksepp, 1981). Given the relatively short period of time in which playfulness is seen in rats, the additional 10 days of play deprivation in the LDB study (an additional 5 days before and 5 days after the SDB deprivation period) represents an appreciable expansion in play deprivation time as compared to the SDB study, while maintaining specificity to the time period in which play is the predominant social behavior.

### Anxiety-like behavior

To determine whether juvenile play deprivation affected anxiety-like behavior later in life, we conducted two tests of anxiety-like behavior. First, we assessed performance on the elevated plus maze at P47, one week after BARR and ISO animals were returned to standard housing conditions in the SDB study and two days after the return to standard housing in the LDB study. Second, we assessed performance on the open field test at P59. This latter open field test was only conducted in the LDB study, as the SDB animals were exposed to the open field in our initial studies (Figure 1C) and as such it was no longer novel. In both tests, we assessed measures of both anxiety-like behavior and hyperactivity.

#### Elevated plus maze

**Figure 2:**
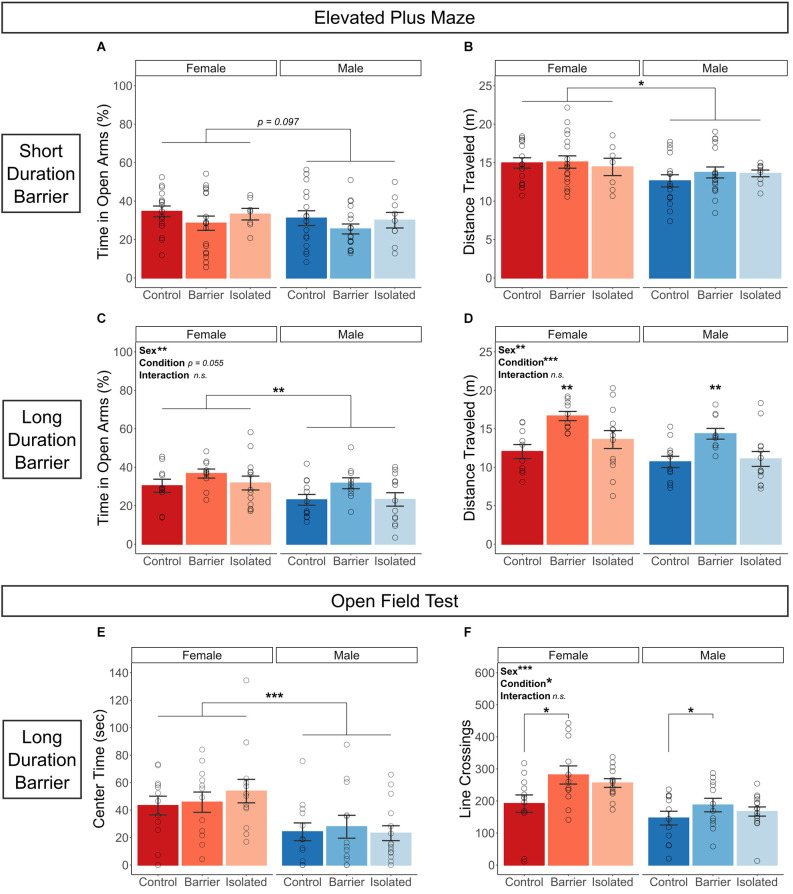
Juvenile play deprivation induces later-life hyperactivity but has no effect on anxiety-like behavior; however, males exhibit more anxiety like behavior than females. Percentage of time spent in the open arms **(A)** and distance traveled **(B)** in an elevated plus maze on P47 in the short duration barrier study, with the same measures shown for the long duration barrier study **(C,D)**. In **(D)**, **indicates BARR animals traveled significantly farther than both CTRL and ISO animals, with *p* < 0.01. An open field test was also conducted at P59 in the long duration barrier experiment, with center time (in seconds) and the number of gridline crossings shown in **(E,F)**, respectively. In **(F)**, *indicates CTRL animals exhibited significantly fewer line crossings than BARR animals with *p* < 0.05. Bars indicate group means ± SEM, and open circles represent data from individual rats. *p < 0.05, **p < 0.01, ***p < 0.001, *n* = 10–15 per group.

In both the SDB and LDB studies, we found no effect of housing condition on time in the open arms (Figures 2A,C). However, replicating other studies (Johnston and File, 1991; Knight et al., 2021), we observed a small but significant sex difference in the LDB animals with a trending effect in the SDB animals (*p* = 0.007 and 0.097, respectively) whereby males spent significantly less time in the open arms of the maze. Mirroring these findings, we observed a significant main effect of sex in the distance traveled in the maze in both studies (SDB: *p* = 0.034; LDB: *p* = 0.005; Figures 2B,D), with males exploring the maze significantly less than females. In the LDB animals, we also observed a main effect of condition (*p* < 0.001) on distance traveled. *Post hoc* analysis indicated that BARR animals of both sexes were significantly more active in the maze than both CTRL (*p* < 0.001) and ISO (*p* = 0.003) animals.

#### Open field test

Similar to performance on the elevated plus maze, we found that males across the three conditions exhibited more anxiety-like behavior, as they spent significantly less time in the center of the open field arena than females (*p* < 0.001; Figure 2E). Additionally, we observed an increase in hyperactivity in animals deprived of play (Figure 2F). *Post hoc* analysis following a significant main effect of both sex (*p* < 0.001) and condition (*p* = 0.013) on the number of gridline crossings indicated that CTRL animals of both sexes were significantly less locomotive than BARR animals (*p* = 0.01), with a trending effect when compared to ISO animals as well (*p* = 0.117). As before, females across conditions were also more locomotive on this test than males.

### Novel object recognition

**Figure 3:**
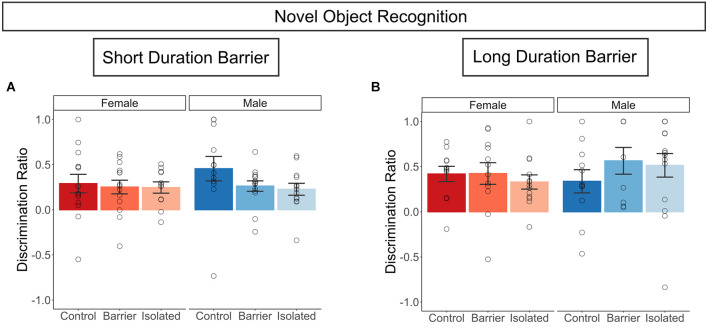
Juvenile play deprivation has no effect on novel object memory in adulthood. Discrimination ratio (**A,B**, calculated as the time spent investigating the novel object minus that of the familiar object, divided by the total time spent investigating either object), in the short and long duration barrier studies, respectively, at P60. Bars indicate group means ± SEM, and open circles represent data from individual rats. *n* = 9–15 per group.

The performance of animals in both the SDB and LDB studies on this task suggested intact object memory within our retention interval (1 h), as evidenced by a discrimination ratio (DR) significantly different from chance (DR = 0; Figures 3A,B) for all six groups. However, there was no effect of sex or housing condition on the discrimination ratio, indicating no difference in object memory.

### Sex behavior

#### Female sex behavior

**Figure 4:**
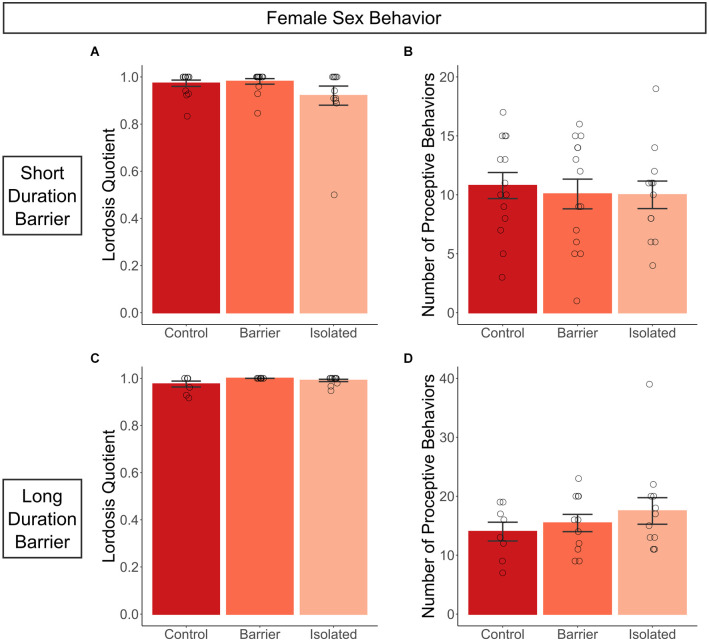
Juvenile play deprivation has no effect on copulatory behavior in adult females. Lordosis quotient **(A)**, the number of lordoses exhibited by the female divided by the total number of mounts by the stimulus male, and the number of proceptive behaviors **(B)**, the total number of hops, darts, and solicitations, exhibited in a 10-min test with a sexually experienced adult male stimulus rat on P63 for the short duration barrier study, with the same measures shown for the long duration barrier study in **(C,D)**. Bars indicate group means ± SEM, and open circles represent data from individual rats. *n* = 7–14 per group.

As described further in the materials and methods section, females in the SDB study were hormonally primed and assessed for copulatory behavior with a sexually experienced adult male rat. We observed no effect of juvenile housing condition on female sexual behavior, as there were no differences in receptivity (lordosis quotient, Figure 4A) or proceptivity (number of proceptive behaviors, Figure 4B) across groups. To ensure the artificial hormonal priming was not obscuring a potential deficit, we allowed for natural cycling in the LDB study, conducting the behavioral assay when female subjects were in proestrus and thus sexually receptive. Even in naturally cycling conditions, we again observed no effect of play deprivation on either sexual receptivity or proceptivity (Figures 4C,D).

#### Male sex behavior

**Figure 5:**
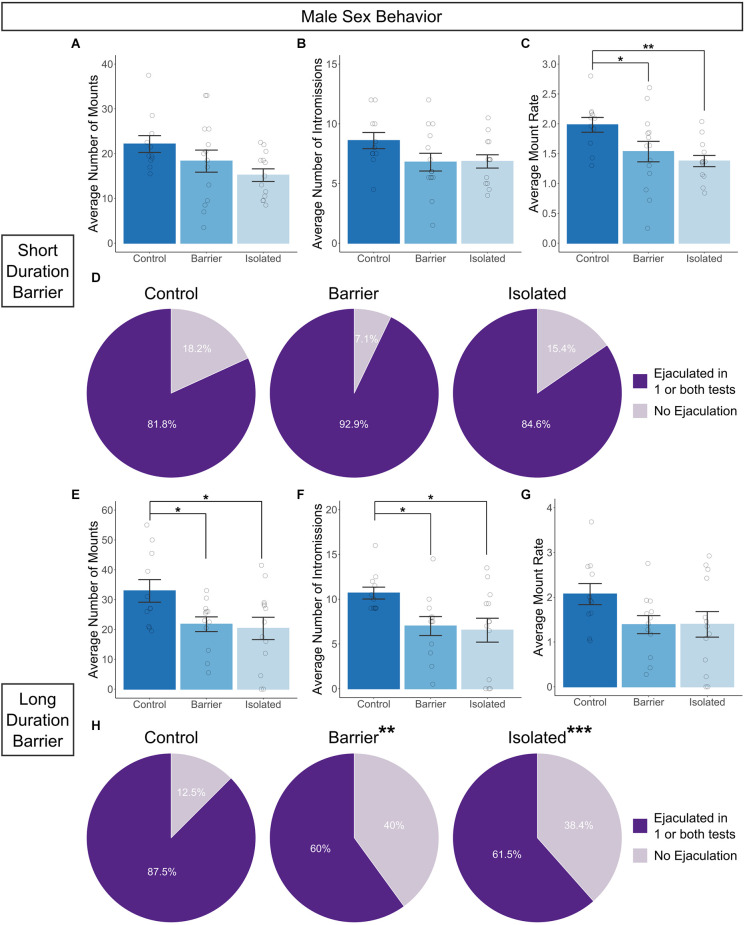
Juvenile play deprivation induces deficits in adult male copulatory behavior. Quantification of the average number of mounts **(A)**, intromissions **(B)**, and the mount rate **(C)**, (the total number of all mounts, intromissions, and ejaculations divided by the active time, multiplied by 60 to indicate the number of mounts per minute) across two 10-min tests with a hormonally primed adult female stimulus rat on P63 and P70 in the short duration barrier experiment. The same measures are shown in **(E–G)** for the long duration barrier experiment. Panels **(D,H)** indicate the proportion of animals in each group which ejaculated on one or both tests. Bars indicate group means ± SEM, and open circles represent data from individual rats. *p < 0.05, **p < 0.01, *n* = 8–14 per group. In **(H)**, ** and ***indicate that the proportion of animals which ejaculated is significantly different (**p < 0.01, ***p < 0.001) from 90%, the amount expected for control animals based on historical data.

In contrast to females, there was a significant impairment in sexual behavior in adult males prevented from playing as juveniles. To account for potential changes in performance due to experience, male sex behavior testing was conducted twice, one week apart on P63 and P70, and values were averaged by subject. We observed either significant or trending effects on the average number of mounts (SDB: *p* = 0.075; LDB: *p* = 0.029; Figures 5A,E) and the average number of intromissions (SDB: *p* = 0.13; LDB: *p* = 0.016; Figures 5B,F) in both the SDB and LDB animals, with BARR and ISO males exhibiting an often stepwise decrease in the numbers of both sexual behaviors compared to CTRL males. This resulted in a significant reduction in the average mount rate (the total number of mounts, intromissions, and ejaculations divided by the active time) in the SDB animals (*p* = 0.014; Figure 5C) which was also trending in the LDB animals (*p* = 0.151; Figure 5G). *Post hoc* analysis of data from the SDB animals revealed both BARR (*p* = 0.03) and ISO (*p* = 0.005) males had a significantly lower mount rate than their CTRL counterparts.

We also assessed the proportion of animals that ejaculated across the two tests of sexual behavior. It is well known in the field of rodent sexual behavior that around 10% of all sexually mature male rats will be “duds” (otherwise known as “sexually sluggish”), meaning that they do not ejaculate in the course of a 20–30 min assay with a sexually receptive female (Dewsbury, 1972; Pattij et al., 2005; Portillo et al., 2006). To determine if play deprivation affected the incidence of “duds”, we calculated the proportion of animals in each group that ejaculated in one or both of the 20-min sex behavior tests, compared to the proportion that did not ejaculate in either test. We then conducted individual chi-square goodness of fit tests comparing the distribution of each group (Male CTRL, BARR, and ISO) to the expected distribution (90% ejaculators, 10% non-ejaculators/“duds”). In the SDB animals, the distribution of ejaculators vs. non-ejaculators did not significantly differ from the expected distribution across any of the three groups (Figure 5D). In the LDB animals, CTRL males also did not show a significant difference (Figure 5H). However, the distribution of ejaculators vs. non-ejaculators in BARR (*p* = 0.002) and ISO (*p* < 0.001) males significantly differed from the expected distribution. In both groups, only ~60% of animals ejaculated in one or both tests, while ~40% were “duds”. Together, these studies indicate that juvenile play deprivation impairs both the number/rate of sexual behaviors (SDB and LDB) and the proportion of animals which successfully ejaculate (LDB) in adult males, a notable finding given the paramount importance sexual behavior plays in reproductive fitness.

### Social preference

**Figure 6:**
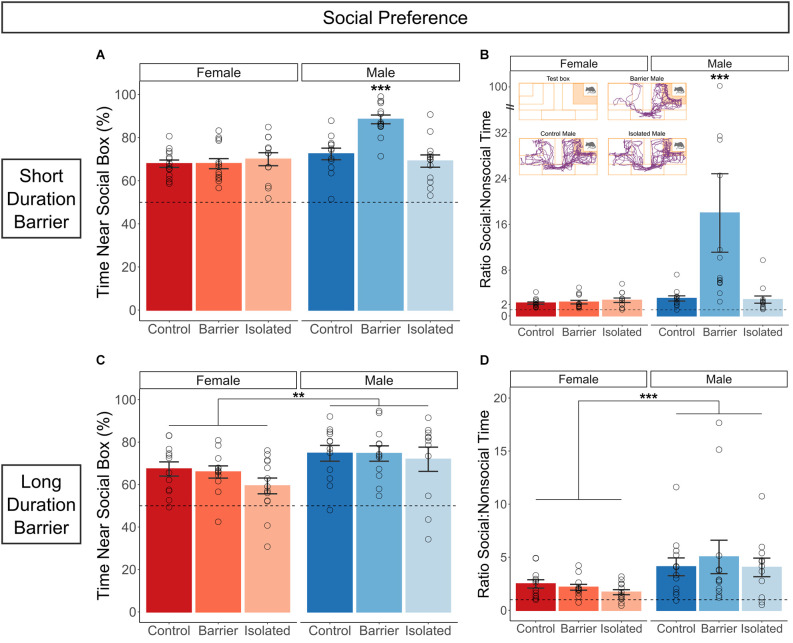
Juvenile play deprivation, but not full social isolation, induces hypersociability in adult males in the short but not the long duration barrier study. Quantification of the percentage **(A)** and ratio **(B)** of time spent near the social stimulus box compared to time spent near the nonsocial, empty stimulus box in the short duration barrier experiment. In **(A,B)**, ***indicates male BARR animals significantly differed from all other groups on both measures, with *p* < 0.001. The inset in **(B)** shows representative traces from CTRL, BARR, and ISO males. The same measures are shown in **(C,D)** for the long duration barrier study. Bars indicate group means ± SEM, and open circles represent data from individual rats. **p < 0.01, ***p < 0.001, *n* = 11–14 per group.

In both the SDB and LDB studies, all six groups demonstrated significant social preference compared to chance (50%), exhibiting a much higher percentage of time interacting closely with the social stimulus box compared to the empty stimulus box. In the SDB study, we additionally observed a significant sex × condition interaction on the percent of time spent near the social stimulus box (*p* < 0.001; Figure 6A; ratio data and representative traces shown in Figure 6B). *Post hoc* analysis indicated that male BARR animals had a significantly higher social preference compared to all other groups, including male CTRL (*p* < 0.001) and male ISO (*p* < 0.001) animals. This appeared to be a true difference in sociability not affected by differences in overall exploration time, as there was no effect of condition on the time spent near either stimulus box (Supplementary Figure 1A). However, while a small but significant (*p* = 0.003) sex difference in the percentage of time near the social box was detected in the LDB study (Figure 6C; ratio data shown in Figure 6D), with males showing increased social preference relative to females, there was no effect of juvenile housing condition.

### Sex preference

**Figure 7:**
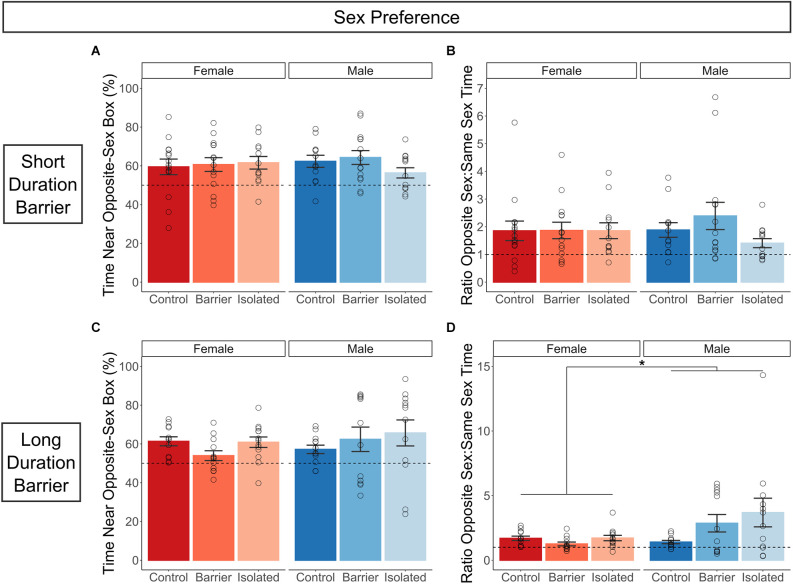
Juvenile play deprivation has no effect on sex preference in adulthood. Quantification of the percentage **(A)** and ratio **(B)** of time spent near the opposite-sex stimulus box compared to time spent near the same-sex stimulus box in the short duration barrier study. The same measures are shown in **(C,D)** for the long duration barrier study. Bars indicate group means ± SEM, and open circles represent data from individual rats. *p < 0.05, *n* = 12–14 per group.

In both the SDB and LDB studies, all six groups demonstrated significant opposite-sex preference as compared to chance (50%), with the exception of the two BARR groups in the LDB study, in which the one-sample *t*-test vs. chance was trending (Male BARR: *p* = 0.075; Female BARR: *p* = 0.148). However, no significant differences were detected as a result of condition on the percent of time spent near the opposite-sex stimulus box (Figures 7A,C), indicating sex preference in adulthood is unaffected by juvenile play deprivation. While there were no sex differences on this measure in either the SDB or LDB animals, a main effect of sex was detected when considering the data as a ratio of the time spent near the opposite sex compared to the same-sex stimulus box in the LDB animals (Figure 7D) but not the SDB animals (Figure 7B), with males exhibiting stronger opposite-sex preference than females.

### Social recognition

**Figure 8:**
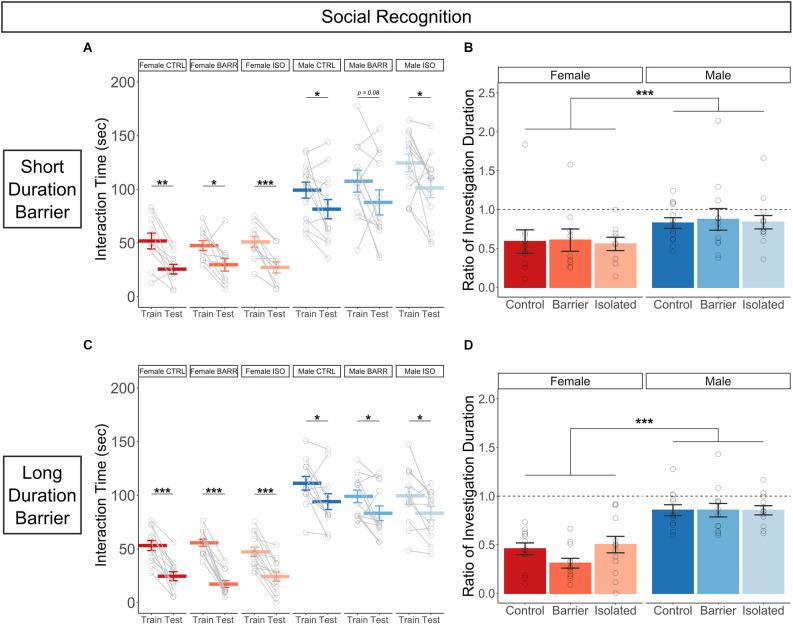
While juvenile play deprivation has no effect, there are sex differences in social recognition in adulthood. Quantification of the amount of time spent interacting with a stimulus rat on both the Train (first trial in which the stimulus rat is novel) and the Test (second trial following a 30 min inter-trial interval in which the stimulus rat is now familiar) trials **(A)** and the ratio of interaction time on the Test trial compared to that of the Train trial **(B)** for the short duration barrier study. The same measures are shown in **(C,D)** for the long duration barrier study. Bars indicate group means ± SEM, and open circles represent data from individual rats. *p < 0.05, **p < 0.01, ***p < 0.001, *n* = 9–13 per group.

In the social recognition assay, subjects were assessed for the amount of time spent interacting with a novel stimulus animal (“Train” trial) compared to the amount of time spent interacting with that same (now familiar) animal on a second trial (“Test” trial) after a 30 min inter-trial interval (Lemaire, 2003). In both the SDB and LDB studies, all six groups demonstrated social recognition (Figures 8A,C), as their interaction time on the Test trial was significantly decreased compared to that of the Train trial, with the exception of the Male BARR group in the SDB study, which was trending (*p* = 0.085). This decreased interaction time appeared to represent true social recognition, as opposed to an artifactual decrease as a result of being assessed in a second consecutive test, as there was no difference in interaction time between the Train and Test trials for any of the six SDB or LDB groups in a control experiment in which subjects received a novel stimulus animal in both trials (Supplementary Figure 2). Interestingly, we detected a strong sex difference in the ratio of investigation duration, with males exhibiting ratios closer to 1, indicating less intact social recognition (SDB: *p* < 0.001; LDB: *p* < 0.001; Figures 8B,D); this followed a sex difference we observed in the overall interaction times, whereby males spent much more time interacting with the stimulus animal across both trials than females did. However, there was no effect of juvenile housing condition, indicating play deprivation has no effect on later-life social recognition.

### Resident intruder assay

**Figure 9:**
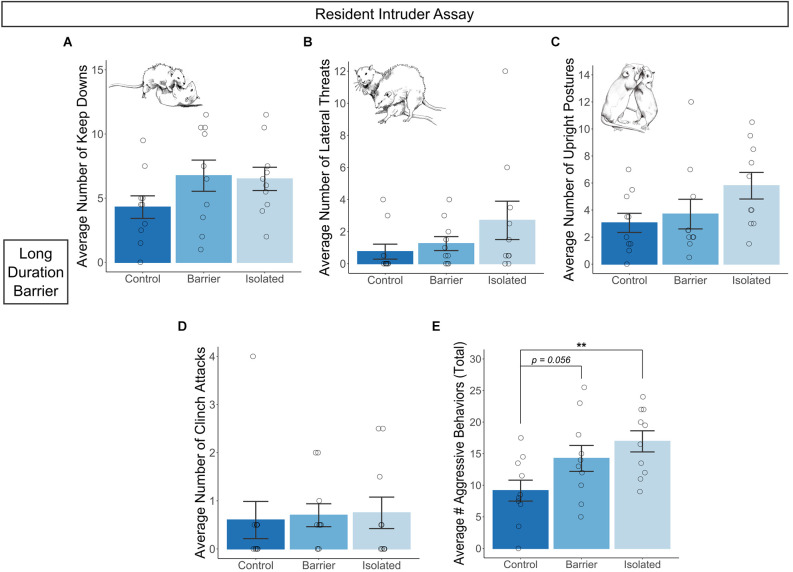
Juvenile play deprivation increases adult aggressive behavior in males. Quantification of the average number of keep downs **(A)**, lateral threats **(B)**, upright postures **(C)**, and clinch attacks **(D)** exhibited by each resident male subject towards a novel intruding stimulus male in two 15-min tests in the long duration barrier experiment. Cartoon insets in **(A–C)** depict the assessed behavior in question. The average number of total aggressive behaviors is shown in **(E)**. Bars indicate group means ± SEM, and open circles represent data from individual rats. **p < 0.01, *n* = 10 per group.

In the LDB study, we conducted two tests of the resident intruder assay using male subjects, separated by a 30 min inter-trial interval with a novel intruder rat each time. We scored various aggressive behaviors as described in Koolhaas et al. (2013) and averaged values by subject. We detected trending effects of condition on the average number of keep downs (*p* = 0.189; Figure 9A), lateral threats (*p* = 0.13; Figure 9B), and upright postures (*p* = 0.088; Figure 9C), but no difference in the number of clinch attacks (Figure 9D). Summing these behaviors together, we calculated the average number of total aggressive behaviors exhibited by subjects across the two tests (Figure 9E) and observed a significant main effect of condition on this measure (*p* = 0.016). *Post hoc* analysis indicated CTRL males exhibited significantly fewer total aggressive behaviors than ISO males (*p* = 0.005), with a trending effect when compared to BARR males (*p* = 0.056), demonstrating that juvenile play deprivation significantly increased aggressiveness in adulthood.

### Empathy/prosocial helping behavior

**Figure 10:**
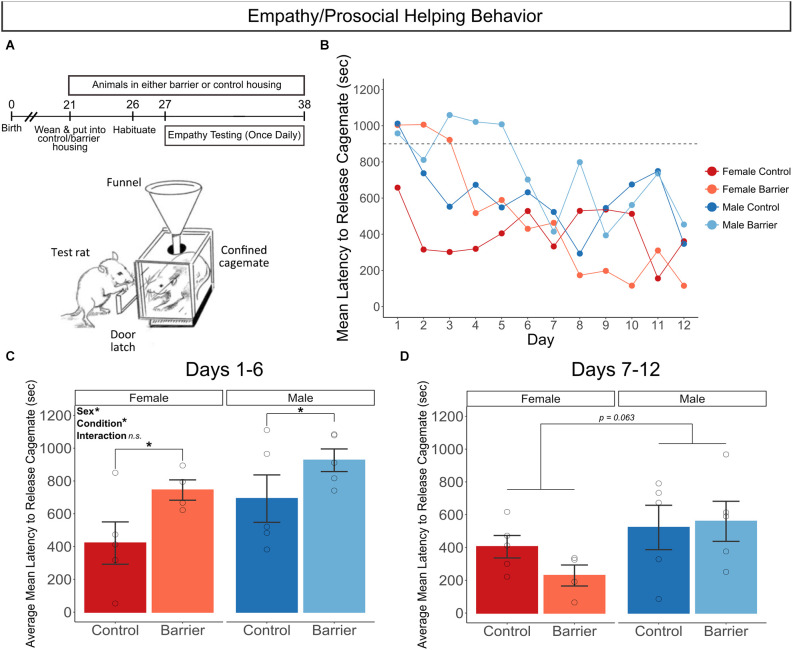
Juvenile play deprivation impairs performance on a test of empathy/prosocial helping behavior. **(A)** Timeline of experiment and cartoon of the prosocial behavior test apparatus, showing the test rat opening the door latch to release its cagemate confined in the box. Quantification of the mean latency (in seconds) to release cagemate across the 12 days of testing **(B)**, with dashed line at 900 s indicating when the door latch was loosened by the experimenter if the test animal had not already released the confined cagemate. Quantification of the average mean latency to release cagemate across the first 6 **(C)** and the last 6 **(D)** days of the test procedure. Bars indicate group means ± SEM, and open circles represent data from individual rats. *p < 0.05, *n* = 4–5 per group.

In a separate experiment from the SDB and LDB studies, we assessed empathy using a test of prosocial helping behavior in animals acutely deprived of play starting a week before and continuing through the 12 days of testing. Rats of both sexes were housed in CTRL or BARR conditions starting on P21 and remained in these conditions until and throughout testing, which began on P27 and lasted until P38 (Figure 10A). In this assay, test subjects are evaluated for whether and how quickly they release their sex- and condition-matched sibling cagemate from a confinement box over the 12 days of repeated daily testing (Figure 10B). Animals deprived of play exhibited significantly longer latencies to free their cagemates on the first six days of the task compared to controls (*p* = 0.027; Figure 10C). The performance of play-deprived animals improved to control levels by the second half of testing, as there was no effect of condition on this measure on days 7–12 of the assay (Figure 10D). There was also a sex difference in this task in both stages of testing, with males on average exhibiting significantly longer latencies to release their cagemates in the early stage of testing (Days 1–6; *p* = 0.046) with a trending effect in the late stage of testing (Days 7–12; *p* = 0.063).

### Factor analysis

Upon completing our analyses, we wondered if any larger patterns or categories of later-life behaviors were specifically altered by play deprivation in the juvenile period. To investigate this, we performed factor analysis, a method for assessing observed variables and their variances to determine whether there are latent constructs (“factors”) that explain the patterns of phenotypes seen in a particular dataset. We conducted this analysis on the SDB and LDB results separately because we were interested in whether the analysis would pull out factors that were similar or different across the two experiments. As is standard for this method, we conducted this analysis on various outcomes from the battery of behavioral experiments, focusing on those most strongly correlated and thus appropriate for inclusion in factor analysis (see “Materials and methods” Section). Consequently, we conducted these analyses on only the male data, as the female data in both the SDB and LDB studies was deemed insufficiently correlated and therefore inappropriate for factor analysis based on the Kaiser-Meyer-Olkin Measure of Sampling Adequacy (MSA; below 0.5 for both studies) and Bartlett’s sphericity test (*p*-value above 0.05 for both studies).

**Figure 11:**
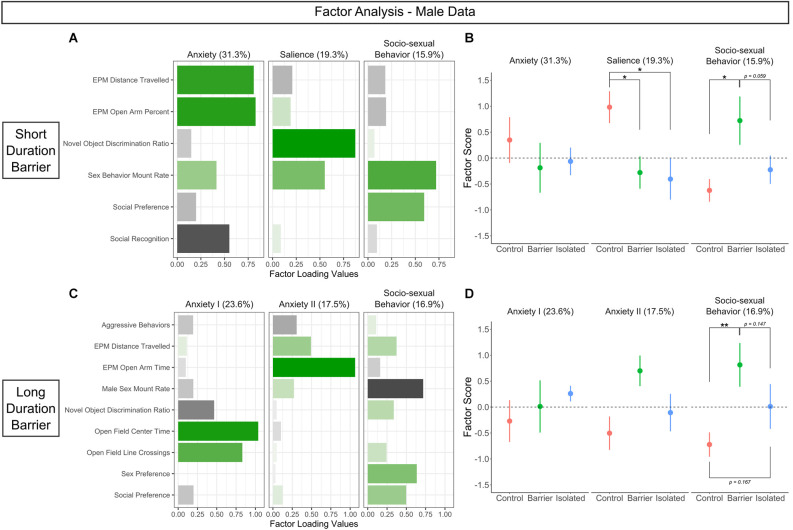
Factor analysis indicates that juvenile play deprivation preferentially affects socio-sexual behavior in adult males. **(A,C)** Behavior factor loadings for each named factor for the short and long duration barrier studies, respectively. Longer, more saturated bars indicate stronger loadings, with green denoting positive loading and black indicating negative loading. **(B,D)** Loading scores for CTRL, BARR, and ISO males plotted for each factor for the short and long duration barrier studies, respectively. The values in parentheses represent the percentage of total variance accounted for by each factor. Filled circles indicate group means ± SEM. *p < 0.05, **p < 0.01, *n* = 6–9 per group.

In the SDB study, six behavioral measures were deemed appropriate for inclusion in our analysis. We identified three factors to be sufficient to explain the data, accounting for 66.5% of the total variance (Figure 11A). The first factor (31.3% of total variance) contained positive loadings for distance traveled and percent of the time in the open arms of the elevated plus maze, with negative loading for social recognition. As these enriched behaviors generally reflected measures of a latent anxiety-like phenotype, we renamed it accordingly (“Anxiety”). The second factor (19.3% of total variance) contained positive loadings for discrimination ratio on the novel object recognition task and sex behavior mount rate, both phenotypes that require recognition of the salience of a given stimulus; thus, we renamed the factor as such (“Salience”). Finally, the third factor (15.9% of total variance) contained positive loadings for sex behavior mount rate and social preference, two tests within the socio-sexual domain (hence titled “Socio-sexual behavior”).

In the LDB study, nine behavioral measures were deemed appropriate for inclusion. From this analysis, we again identified three factors to be sufficient to explain the data, accounting for 58% of the total variance (Figure 11C). The first two factors appeared to reflect a latent anxiety-like phenotype, separating based on the specific anxiety-like behavior test in question. Factor one (23.6% of total variance) contained strong positive loadings for center time and line crossings in the open field test, with negative loading for novel object discrimination ratio, while factor two (17.5% of total variance) contained strong positive loadings for distance traveled and open arm time on the elevated plus maze. As such, we renamed the factors as “Anxiety I” and “Anxiety II”, respectively. Similar to the SDB study, the third factor (16.9% of total variance; hence titled “Socio-sexual behavior”) contained loadings for various socio-sexual behaviors: positive loadings for sex preference and social preference and negative loading for sex behavior mount rate.

To determine whether the expression of these factors differed across our three groups, we calculated factor scores for each subject (SDB: Figure 11B; LDB: Figure 11D). No significant differences were observed for the Anxiety-related factors in either the SDB or the LDB study. However, in the SDB animals, there was a significant effect of condition on the Salience factor (*p* = 0.034). *Post hoc* analysis detected a significant difference between CTRL animals and those deprived of play on this factor (BARR: *p* = 0.029; ISO: *p* = 0.015), with CTRL males exhibiting higher positive scores on this Salience factor than both play-deprived groups. Most notably, we also detected a significant main effect of condition on the Socio-sexual Behavior factor in both the SDB (*p* = 0.044) and LDB (*p* = 0.03) animals. *Post hoc* analyses indicated BARR males had a significantly higher positive score for this factor than CTRL males in both studies (SDB: *p* = 0.019; LDB: *p* = 0.008), and there were trending effects when compared to ISO males in both studies as well (SDB: *p* = 0.059; LDB: *p* = 0.147). Together, these analyses suggest that socio-sexual behavior is a distinct category of later-life behavior specifically sensitive to play deprivation in juvenile males.

## Discussion

Here, we report persistent alterations in the expression of various adult behaviors following juvenile play deprivation in rats. We demonstrate that social play experience is critical for the development of appropriate socio-sexual behavior in males, as males prevented from playing as juveniles exhibited altered sexual behavior, sociability, and aggressive behavior. These effects are complex and pervasive, seen weeks to months after all animals were returned to group housing, and impact reproductively critical behaviors. In females, however, play is more dispensable. While rats of both sexes were hyperactive shortly after play deprivation and displayed impairments in a test of empathy motivation when acutely prevented from playing, we observed no effects in females on any other test in the diverse battery we conducted.

Given the importance for reproductive fitness, the most striking of our findings is the lasting effect of play deprivation on male sexual behavior. Males prevented from playing for as little as 2 weeks (in the SDB study) showed a significant reduction in mount rate across two tests of sexual behavior. This was seen in both BARR and ISO males, indicating that the lack of play, not the lack of social interaction writ large, was the critical driver of this impairment. Moreover, this impairment did not seem to represent a simple delay in the learning process, but rather a persistent reduction in the rate of copulatory behavior; while we present the data here as an average, the deficit in BARR and ISO males was evident across both successive tests. Furthermore, males prevented from playing for as little as 3.5 weeks (in the LDB study) also exhibited deficits in ejaculation. Around 40% of BARR and ISO males did not ejaculate in either test of copulatory behavior, a significant deviation from what was seen in CTRL males (12.5%) and from the expected distribution of non-ejaculators (~10%) based on decades of research on male rat sexual behavior (Dewsbury, 1972; Pattij et al., 2005; Portillo et al., 2006). Remarkably, these impairments were close to the magnitude seen in other studies in which male copulatory behavior is altered by more substantial pharmacological or genetic manipulations, despite the fact that our manipulation merely prevented juveniles from playing for a short window of time. For example, the decrease in the number of mounts we observed was of similar magnitude to that seen in males treated with a cyclooxygenase-2 inhibitor for 2 weeks during the perinatal sensitive period (Amateau and McCarthy, 2004), and the reduction in the percentage of animals which successfully ejaculated we observed was similar to that seen in males in which both the androgen receptor and estrogen receptor alpha were genetically deleted (Trouillet et al., 2022).

We also found that play deprivation induces hypersociability in adulthood. In the SDB study, BARR males exhibited social preference that was significantly higher than that seen in all other groups. This hypersocial phenotype was seen across multiple SDB cohorts, and the effect size was substantial (Cohen’s *d* ranging between 1.9 and 2.9 for all pairwise comparisons between Male BARR and the other groups for percent of time near the social box; Figure 6A). However, there was no effect on social preference in the LDB animals. This differential effect in the SDB compared to the LDB study may be due to an as-yet-unidentified sensitive period for social development around the time of weaning. Work from Hol et al. (1999) demonstrated that social isolation from P22–28, but not P29–35, decreases sociability in adulthood in male rats. Isolation across the full window, from P22–35, also decreased sociability. This finding was mirrored by a study in females which found that isolation from P19–70 also decreases sociability (Hermes et al., 2011). In contrast, isolation starting later in adolescence, from P28–70, was shown to produce hypersociability in both sexes, although this study was conducted in mice (Rivera-Irizarry et al., 2020). Looking at the intersection of these studies, it is possible that two distinct sensitive periods exist, perhaps governed by the maturation of distinct sets of social circuitry: one window from ~P19–28, in which the lack of play experience causes hyposociability, and another starting at ~P35, in which the lack of play experience causes hypersociability. In this view, it is possible we observed no effect on sociability in the LDB study as the play deprivation spanned across those windows (from P21–45), canceling out any effects. It is also possible that the additional stress caused by full isolation may alter the direction and magnitude of these effects, given ours were seen only in BARR, but not ISO, males. Future studies should more closely investigate the possibility of these distinct sensitive periods and whether they may differ based on sex, given the hypersocial phenotype we observed was seen only in males.

One of many theories regarding the function of social play is the “play-as-practice” theory, which argues that the individual behaviors constituting social play (e.g., pounces, pins, and boxing behaviors in rats) resemble adult agonistic behaviors for a reason: because play serves as practice for later-life aggressive encounters (Fagen, 1981; Smith, 1997). This theory has received criticism for various reasons, primarily that there are several key differences in the microstructure of juvenile play-fighting and adult aggressive behaviors, thus hindering play’s applicability as a means of physical “practice” (Pellis and Pellis, 1987). We here provide further evidence against the play-as-practice theory, as we demonstrate that juvenile play deprivation increases aggressive behavior in adult males, not decreases as a straightforward interpretation of the theory would predict. Nonetheless, a more complex interpretation of the theory may be better justified. While it seems evident that play does not serve as simple practice for the motor skills needed for later-life agonistic behavior, perhaps what is gained from play is experience in its reciprocal nature. This reciprocity—necessitating monitoring one’s own actions and those of a partner, recognizing social signals indicating the partner’s responses and intentions, and adjusting one’s actions appropriately to maintain the back-and-forth of the play bout—is a fundamental feature of social play (Pellis and Pellis, 2017), so play may serve to provide experience in these social skills, allowing for the maturation of brain circuitry to the same effect. Animals deprived of play may lack this experience, thereby hindering their ability to appropriately recognize and/or respond to social signals to de-escalate an agonistic encounter, resulting in increased aggression as we observed. Indeed, recent work in *Drosophila* identified a transcriptional regulator believed to govern this same effect, suppressing aggression in a social experience-dependent manner (Ishii et al., 2022), and long-term post-weaning social isolation has been shown to induce qualitative differences in the attack patterns of male rats (Toth et al., 2011).

We also conducted a test of prosocial motivation in animals acutely deprived of play starting a week before and continuing throughout the 12 days of testing. BARR animals of both sexes performed worse on this task, taking significantly longer to free their distressed cagemate from a confinement box on the first 6 days of testing. This impairment was resolved by the latter half of testing, with the performance of play-deprived animals improving to that seen in controls on days 7–12 of the paradigm. This finding may serve as another example of the concept that play aids in developing the ability to appropriately sense and respond to social signals. In this view, BARR animals may perform worse on the empathy test due to a deficit in their perception of and/or responsiveness to the social signals generated by their distressed cagemate. This impairment may have improved over time due to the increased social experience afforded as part of the test (while animals were allowed only 10 s to interact with their cagemate following release from the confinement box, this social experience may have accumulated over time), or because animals had sufficient experience with the paradigm by the latter half of testing such that it allowed BARR animals to “catch up” to controls. We also observed a sex difference in this assay: males generally performed worse than females, as they exhibited a significantly higher latency to release their cagemate on average in the first half of testing. This too may speak to the heightened importance of play experience in males compared to females, given even at baseline males are less successful on this socially-driven task.

Importantly, we saw no effects of play deprivation on any other assessed behavior, with the exception of a small but significant effect on locomotion shortly after animals were returned to standard group housing. Our findings in which we observed play deprivation had no effect—on anxiety-like behavior, object memory, sex preference, and social recognition in both sexes and on sex behavior and social preference in females—are just as informative as our findings in which we did observe an effect, in that they show what play experience does *not* seem to be important for. The effects of play deprivation (and therefore, the importance of play itself) seem to preferentially center around socio-sexual behaviors in adulthood, a concept further supported by our factor analyses, which in an independent and unbiased manner identified factors in both the SDB and LDB studies that represent socio-sexual behavior and significantly differed in control and play-deprived males.

This specificity to socio-sexual behavior makes sense, given play is itself a social behavior and involves the activity of many regions in the social circuitry, such as the medial amygdala, lateral septum, and hypothalamus (summarized in Siviy, 2016 and VanRyzin et al., 2020a). These regions have distinct roles in socio-sexual behavior in adulthood as well, often driven by distinct, separable cell type-specific and projection-specific mechanisms (Unger et al., 2015; Wong et al., 2016; Ishii et al., 2017; Kohl et al., 2018). The maturation of these cell subtypes and/or projections, then, may be modulated in an experience-dependent (and, potentially, sex-dependent) manner based on playfulness. Like the importance of visual experience for the development of ocular dominance in V1 neurons (Wiesel and Hubel, 1963) or somatosensory experience for dendritic spine dynamics in the barrel cortex (Lendvai et al., 2000), play experience may be fundamental to the developmental firing and wiring of individual cells and projections that establishes appropriate adult circuit organization. However, the fact that the impairments we observed following play deprivation were generally specific to males begs the question: in this view, what, if anything, governs social circuit maturation in females? Does social experience (play or non-play) have any role? In our initial studies, we found that the additional non-play social experience provided by the BARR vs. the ISO housing condition is sufficient to decrease play motivation in females but not males (Figure 1C), suggesting that play itself is the critical feature driving the motivation to play in males. Perhaps in females, the significance of play is more so the social experience in general, and less so the experience of play itself. Even then, it appears little social experience during the juvenile window is necessary for appropriate social development in females, given we observed few impairments following even full social isolation. Future studies should investigate whether the window for experience-dependent social circuit organization is shifted in females compared to males, or if, perhaps, female social circuit maturation is not experience-dependent at all.

While our BARR manipulation improves upon previous methodology in that it prevents play while allowing for other forms of social interaction, there are nonetheless limitations. BARR animals are also deprived of other tactile non-play social interactions such as social grooming, and there may be other variables such as the difference in physical space allowed by the BARR vs. CTRL and ISO conditions that may affect development. Nevertheless, the convergence of evidence from our study and others using different manipulations (e.g., reducing play by rearing juveniles with an adult or a peer from a less playful strain) points to play experience being the critical driver of the impairments observed (Schneider et al., 2016; Burleson et al., 2016; Pellis et al., 2017; Stark and Pellis, 2020, 2021). Additionally, while females appeared largely resilient to play deprivation across the myriad of behavioral assays we conducted, this may not be true for all traits, or there may be latent differences in our assessed traits not detectable by the paradigms we used to evaluate them. Regardless, this female resilience is notable, supporting our hypothesis that the purpose of play differs across the sexes, at least in the rat.

Embedded within our studies is also a deep assessment of sex differences and similarities across a wide variety of behavioral tests in rats. When possible (i.e., in assays that are not only applicable to animals of one sex), males and females were included in all experiments, all of which were adequately powered to detect sex differences if they were present. We statistically tested sex as a variable, calling a finding a “sex difference” if and only if we detected a significant main effect of sex or a significant interaction. This was as opposed to independently assessing males and females and noting a sex difference if we observed an effect in one sex but not the other, a common misconception of the appropriate way to assess sex differences (Garcia-Sifuentes and Maney, 2021). As the research community works to better address sex as a biological variable (SABV), especially following the implementation of the United States National Institutes of Health’s 2016 policy requiring consideration of SABV in all funded research, we hope this work is useful to behavioral neuroscientists interested in the differences and similarities in male and female rats across various behavioral domains.

Finally, our results are especially relevant in the wake of the COVID-19 pandemic. We began these studies shortly before shutdowns began in early 2020, impacting access to play for millions of children worldwide, and as such our experiments became even more relevant. While our studies were conducted in rats, social play is remarkably similar across the many mammalian species in which it is expressed and has strong face validity in humans. Our results would argue that boys may be more vulnerable to a reduction in access to play than girls. This potential sex difference in the influence of the pandemic on child health and brain development is something that should be monitored as we collectively move forward.

## Data Availability Statement

The raw data supporting the conclusions of this article will be made available by the authors, without undue reservation.

## Ethics Statement

The animal study was reviewed and approved by Institutional Animal Care and Use Committee, University of Maryland School of Medicine.

## Author Contributions

AM and MM designed research and wrote the manuscript. AM, JV, and RF performed research. AM and JV analyzed data. All authors contributed to the article and approved the submitted version.
