# Supplementary material for: Social play experience in juvenile rats is indispensable for appropriate socio-sexual behavior in adulthood in males but not females
Source: Front Behav Neurosci. 2023 Jan 23;16:1076765. doi: 10.3389/fnbeh.2022.1076765 (PMC9899815; doi:10.3389/fnbeh.2022.1076765)
Supplement: Supplementary file 1 [file Data_Sheet_1.DOCX]

Supplementary Material

**
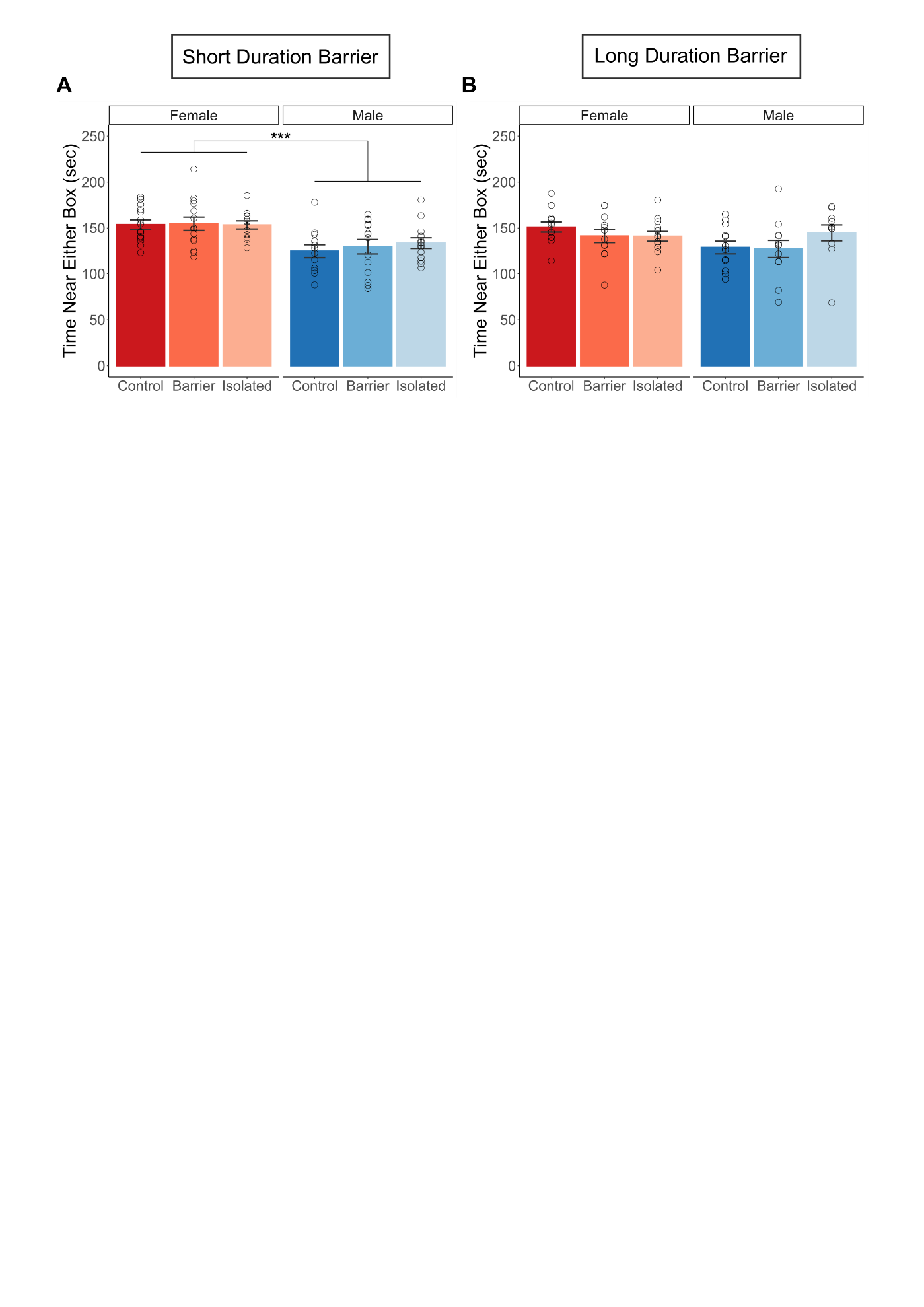
**

**Supplementary Figure 1.** Juvenile play deprivation has no effect on the time spent investigating either box in the social preference test in both the short (***a***) and long duration barrier (***b***) studies. Bars indicate group means ± SEM, and open circles represent data from individual rats. ***p < 0.001, *n* = 11-14 per group.


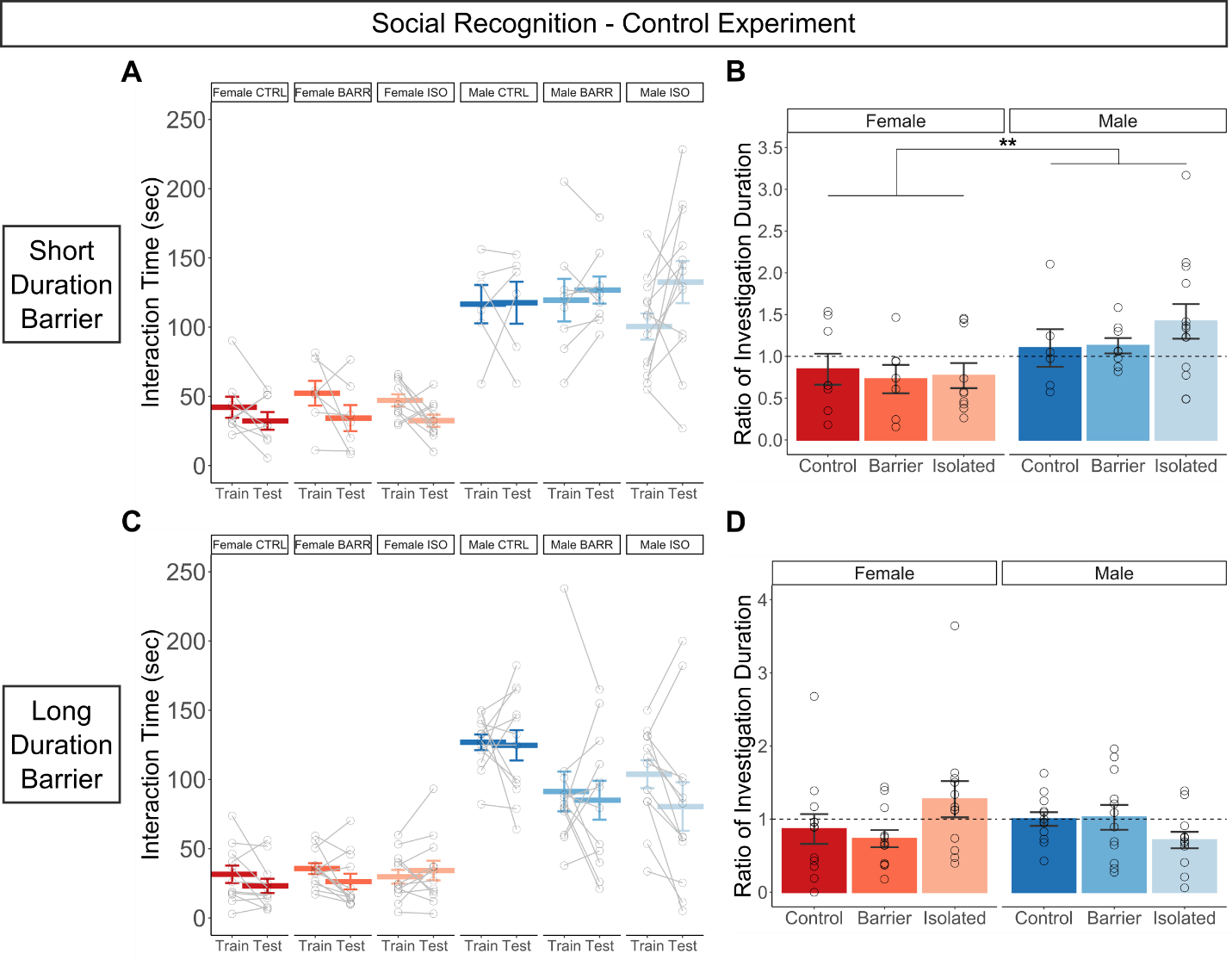


**Supplemental Figure 2.** The assay used to assess social recognition is reliable, as indicated by the lack of a statistical difference in the interaction time in a control experiment in which a novel stimulus animal is used in both the Train and Test trials (as opposed to a familiar stimulus on the Test trial). Quantification of the amount of time spent interacting with the stimulus rat (***a***) and the ratio of interaction time on the Test trial compared to that of the Train trial (***b***) for the short duration barrier study. The same measures are shown in ***c*** and ***d*** for the long duration barrier study. Bars indicate group means ± SEM, and open circles represent data from individual rats. **p < 0.01, *n* = 9-13 per group.

| **Test** | **Data structure** | **Type of test** | **Description of analysis** | **Test value** | **p-value** | **Effect size** |
| --- | --- | --- | --- | --- | --- | --- |
| **Social Preference - SDB** |  |  |  |  |  |  |
| Time Near Either Box | **Normal distribution** | **Two-way ANOVA** | **Effect of sex** | **F(1,73)=21.647** | **<0.001** | **η2=0.231** |
|  | Normal distribution | Two-way ANOVA | Effect of condition | F(2,73)=0.205 | 0.815 | η2=0.006 |
|  | Normal distribution | Two-way ANOVA | Interaction: sex x condition | F(2,73)=0.237 | 0.789 | η2=0.006 |
|  |  |  |  |  |  |  |
|  |  |  |  |  |  |  |
| **Social Preference - LDB** |  |  |  |  |  |  |
| Time Near Either Box | Normal distribution | Two-way ANOVA | Effect of sex | F(1,66)=3.512 | 0.065 | η2=0.049 |
|  | Normal distribution | Two-way ANOVA | Effect of condition | F(2,66)=0.653 | 0.524 | η2=0.019 |
|  | Normal distribution | Two-way ANOVA | Interaction: sex x condition | F(2,66)=1.709 | 0.189 | η2=0.049 |
|  |  |  |  |  |  |  |
| **Social Recognition – Control Experiment - SDB** | |  |  |  |  |  |
| Interaction Time | Normal distribution | Paired *t*-test | Male CTRL - Train vs. Test | *t*(5)=-0.058 | 0.956 | *d*=-0.029 |
|  | Normal distribution | Paired *t*-test | Male BARR - Train vs. Test | *t*(7)=-0.817 | 0.441 | *d*=-0.163 |
|  | Normal distribution | Paired *t*-test | Male ISO - Train vs. Test | *t*(12)=-2.003 | 0.068 | *d*=-0.694 |
|  | Normal distribution | Paired *t*-test | Female CTRL - Train vs. Test | *t*(7)=1.292 | 0.237 | *d*=0.493 |
|  | Normal distribution | Paired *t*-test | Female BARR - Train vs. Test | *t*(6)=1.645 | 0.151 | *d*=0.737 |
|  | Normal distribution | Paired *t*-test | Female ISO - Train vs. Test | *t*(9)=2.128 | 0.062 | *d*=1.062 |
| Ratio of Investigation Duration | **Non-normal** | **Wilcoxon rank-sum test** | **Effect of sex** | ***W*=183** | **0.005** | ***r*=0.392** |
|  | Non-normal | Kruskal-Wallis test: males | Effect of condition | χ2(2)=1.265 | 0.531 | η2(H)=-0.031 |
|  | Non-normal | Kruskal-Wallis test: females | Effect of condition | χ2(2)=0.308 | 0.857 | η2(H)=-0.077 |
|  |  |  |  |  |  |  |
| **Social Recognition - Control Experiment - LDB** | |  |  |  |  |  |
| Interaction Time | Normal distribution | Paired *t*-test | Male CTRL - Train vs. Test | *t*(11)=0.185 | 0.856 | *d*=0.074 |
|  | Normal distribution | Paired *t*-test | Male BARR - Train vs. Test | *t*(11)=0.448 | 0.663 | *d*=0.128 |
|  | Normal distribution | Paired *t*-test | Male ISO - Train vs. Test | *t*(11)=1.975 | 0.074 | *d*=0.395 |
|  | Normal distribution | Paired *t*-test | Female CTRL - Train vs. Test | *t*(11)=1.722 | 0.113 | *d*=0.431 |
|  | Normal distribution | Paired *t*-test | Female BARR - Train vs. Test | *t*(11)=1.839 | 0.093 | *d*=0.543 |
|  | Normal distribution | Paired *t*-test | Female ISO - Train vs. Test | *t*(11)=-0.897 | 0.389 | *d*=-0.199 |
| Ratio of Investigation Duration | Non-normal | Wilcoxon rank-sum test | Effect of sex | *W*=627.5 | 0.822 | *r*=0.027 |
|  | Non-normal | Kruskal-Wallis test: males | Effect of condition | χ2(2)=3.227 | 0.199 | η2(H)=0.037 |
|  | Non-normal | Kruskal-Wallis test: females | Effect of condition | χ2(2)=4.299 | 0.117 | η2(H)=0.07 |

**Table 1.** Summary of statistical parameters for data shown in supplementary figures.
